# Concurrent *Ascaris* infection modulates host immunity resulting in impaired control of *Salmonella* infection in pigs

**DOI:** 10.1128/msphere.00478-24

**Published:** 2024-08-14

**Authors:** Ankur Midha, Larissa Oser, Josephine Schlosser-Brandenburg, Alexandra Laubschat, Robert M. Mugo, Zaneta D. Musimbi, Philipp Höfler, Arkadi Kundik, Rima Hayani, Joshua Adjah, Saskia Groenhagen, Malte Tieke, Luis E. Elizalde-Velázquez, Anja A. Kühl, Robert Klopfleisch, Karsten Tedin, Sebastian Rausch, Susanne Hartmann

**Affiliations:** 1 Department of Veterinary Medicine, Institute of Immunology, Centre for Infection Medicine, Freie Universität Berlin, Berlin, Germany; 2 Charité Universitätsmedizin Berlin, Corporate member of Freie Universität Berlin and Humboldt-Universität zu Berlin, iPATH.Berlin, Core unit of Charité, Campus Benjamin Franklin, Berlin, Germany; 3 Department of Veterinary Medicine, Institute of Veterinary Pathology, Freie Universität Berlin, Berlin, Germany; 4 Department of Veterinary Medicine, Institute of Microbiology and Epizootics, Centre for Infection Medicine, Freie Universität Berlin, Berlin, Germany; University at Buffalo, Buffalo, New York, USA

**Keywords:** helminths, *Ascaris*, *Salmonella*, coinfection, immunomodulation, pig

## Abstract

*Ascaris* is one of the most widespread helminth infections, leading to chronic morbidity in humans and considerable economic losses in pig farming. In addition, pigs are an important reservoir for the zoonotic salmonellosis*,* where pigs can serve as asymptomatic carriers. Here, we investigated the impact of an ongoing *Ascaris* infection on the immune response to *Salmonella* in pigs. We observed higher bacterial burdens in experimentally coinfected pigs compared to pigs infected with *Salmonella* alone. The impaired control of *Salmonella* in the coinfected pigs was associated with repressed interferon gamma responses in the small intestine and with the alternative activation of gut macrophages evident in elevated CD206 expression. *Ascaris* single and coinfection were associated with a rise of CD4^-^CD8α^+^FoxP3^+^ Treg in the lymph nodes draining the small intestine and liver. In addition, macrophages from coinfected pigs showed enhanced susceptibility to *Salmonella* infection *in vitro* and the *Salmonella-*induced monocytosis and tumor necrosis factor alpha production by myeloid cells was repressed in pigs coinfected with *Ascaris*. Hence, our data indicate that acute *Ascaris* infection modulates different immune effector functions with important consequences for the control of tissue-invasive coinfecting pathogens.

**IMPORTANCE:**

In experimentally infected pigs, we show that an ongoing infection with the parasitic worm *Ascaris suum* modulates host immunity, and coinfected pigs have higher *Salmonella* burdens compared to pigs infected with *Salmonella* alone. Both infections are widespread in pig production and the prevalence of *Salmonella* is high in endemic regions of human Ascariasis, indicating that this is a clinically meaningful coinfection. We observed the type 2/regulatory immune response to be induced during an *Ascaris* infection correlates with increased susceptibility of pigs to the concurrent bacterial infection.

## INTRODUCTION

Helminth infections are widespread among humans and animals and occur alongside numerous microbial pathogens (1). *Ascaris lumbricoides* is the most prevalent helminth infection with over 800 million people infected globally (2). The closely related and zoonotic *Ascaris suum* is widespread in pig farming leading to significant production losses (3). Surveys of pig farms in different countries often find high infestation rates with most, if not all, farms impacted (4). *Ascaris* infection follows ingestion of eggs containing infective third-stage larvae (L3) which hatch in the host gut prior to migrating through host tissues by first invading the intestinal barrier, reaching the liver via the portal vein, and passing through the lungs 6–8 days post-infection (dpi) (5). The larvae then penetrate the alveoli, reaching the pharynx to be swallowed and return to the intestine where they reside and develop to L4 primarily in the jejunum from 8 to 10 dpi onwards (5).

Salmonellosis is one of the most commonly reported intestinal infections in the European Union and is caused by serovars of *Salmonella enterica,* a zoonotic, facultative intracellular, food-borne bacterial pathogen (6). The prevalence of nontyphoidal *Salmonella* is high in helminth-endemic areas (7). Pigs are the second most common source of zoonotic *Salmonella* after poultry and *Salmonella* can be detected throughout the pig production chain (8, 9). Infection follows ingestion of contaminated food products, and once arriving in the small intestine, *Salmonella* can traverse the intestinal wall within minutes after invasion of microfold (M) and intestinal epithelial cells (10). Phagocytes, primarily macrophages, take up the invading bacteria and transport them to local gut-associated lymphoid tissues (11, 12). While symptomatic infections are usually accompanied by diarrhea and fever, pigs are often subclinical carriers with studies reporting up to 36% of pigs as asymptomatic shedders (13). In pigs, *Salmonella* can persist within macrophages in lymph nodes for months until market age (14) and the stress of transport can lead to bacterial egress and shedding, leading to increased *Salmonella* loads and contamination of work surfaces and food products (14, 15).

Helminth infections typically induce modified type 2 immune responses, characterized by the release of effector cytokines interleukin (IL-) 4, IL-5, and IL-13 from Th2 cells and type 2 innate lymphoid cells (ILC), and by heightened production of anti-inflammatory IL-10 by regulatory T (Treg) cells. This cytokine profile ensues in type 2 antibody responses, eosinophilia, the accumulation of alternatively activated M2 macrophages, and tissue remodeling including goblet cell hyperplasia designed to harm the migratory larval stages and to expel larval and adult worms from infected intestines (16). Accordingly, *Ascaris*-infected pigs have increased mRNA levels for Th2 and Treg-associated cytokines and there is evidence supporting a role for eosinophils in larval clearance (17–20). By contrast, immunity against *Salmonella* involves mixed type 1/3 responses carried by NK cells, Th1 cells, and Th17 cells and driven by pro-inflammatory cytokines such as tumor necrosis factor-α (TNF-α), IL-12, interferon-γ (IFN-γ), and IL-18, as well as IL-1β and IL17-A which support anti-bacterial responses by monocytes, conventionally activated M1 macrophages, and neutrophils (21–24). Macrophages activated by pro-inflammatory cytokines can defend against intracellular bacteria; however, *Salmonella* can subvert the antimicrobial activity of macrophages and establish a niche within these cells by promoting alternative activation and preventing lysosome fusion while dwelling inside a *Salmonella*-containing vacuole (25–27).

Helminths have been shown to impair responses to bacterial antigens (28–31) and one study has shown increased *Salmonella* burdens during an ongoing helminth infection in mice (32). As such data are lacking in clinically relevant, non-model animals, in this study we asked whether an ongoing *Ascaris* infection impacts immune responses to *Salmonella* in pigs. We show that *Ascaris* infection modulates host immunity and that coinfected pigs have higher *Salmonella* burdens compared to pigs infected with *Salmonella* alone.

## RESULTS

### *Ascaris*-coinfected pigs have elevated *Salmonella* burdens

**Fig 1:**
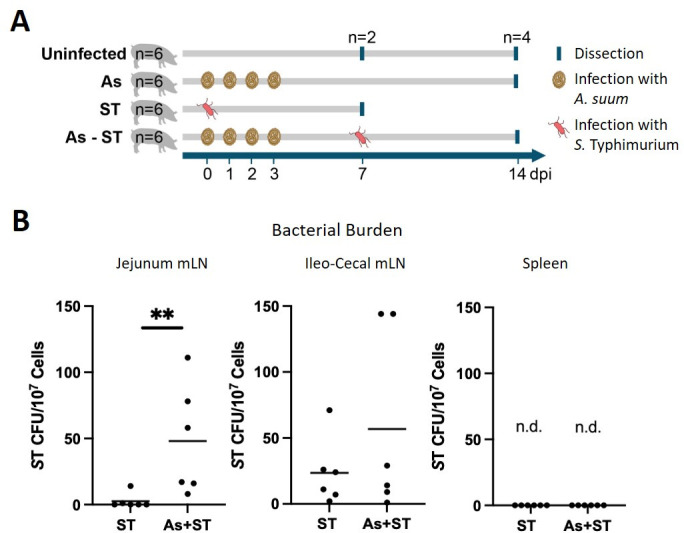
*Ascaris* and *Salmonella* are coinfecting pathogens of slaughterhouse pigs and experimental coinfection shows elevated *Salmonella* burdens. (A) Experimental design. Crossbred 6-week-old pigs (hybrid Landrace and Large White) were left uninfected (Ctrl), experimentally infected with 4 inocula of 2,000 embryonated *A. suum* eggs (As), infected with 10^7^ colony forming units (CFU) *S*. Typhimurium (ST), or infected with both pathogens (As + ST). Pigs were dissected either 14 days post-infection (dpi) with *A. suum* or 7 dpi with *S*. Typhimurium and the same dissection timepoints were maintained in the coinfected group. (B) *S*. Typhimurium CFU was assessed in the mesenteric lymph nodes (mLN) of the jejunum and ileum (ileocecal) and in the spleen; n.d. = not detected. Statistical significance determined by Mann-Whitney test; **, *P* ≤ 0.01.

In an experimental coinfection, we sought to determine whether *Ascaris* infection impacts bacterial burdens in pigs. Commercial weaning pigs were infected with four consecutive inocula of 2000 *A*. *suum* eggs alone (As), 10^7^ CFU *S*. Typhimurium alone (ST), or both pathogens (As + ST) or left uninfected (Ctrl; Fig. 1A). Pigs in the coinfection group were infected first with *A. suum*, and then with *S*. Typhimurium 7 days later. In this experimental design, animals were dissected 14 days after the initial *A. suum* infection and 7 days after infection with *S*. Typhimurium. This time point was chosen as it represents the time when most *Ascaris* larvae have returned to the jejunum following tissue migration (5), and *Salmonella* have been carried into host tissues by phagocytes where they may persist long term (14, 33). Bacterial burdens were assessed locally in mLN of the jejunum and ileum (ileocecal mLN) and in the spleen to assess for systemic dissemination. In the mLN at the primary site of *Salmonella* infection in the ileum, bacterial burdens were not significantly different, although two pigs in the coinfection group exhibited higher bacterial burdens than any of the pigs infected with *Salmonella* alone (Fig. 1B). In the mLN at the site of *Ascaris* infection in the jejunum, pigs infected with *Salmonella* alone were free of bacteria except for one pig while coinfected pigs had significantly elevated bacterial burdens (Fig. 1B). None of the pigs had bacteria in the spleen suggesting that none developed a systemic infection (Fig. 1B). None of the pigs in the trial developed any signs or symptoms of acute salmonellosis and weight gain was not impacted by either infection (Fig. S1). Interestingly, coinfection did not appear to affect tissue pathology. Both *Ascaris* and coinfected pigs demonstrated liver pathology due to migrating larvae, whereas no significant pathology was seen in the lung (Fig. S2). All infected pigs had similar pathology scores in the jejunum and ileum (Fig. S2).

### *Ascaris* infection suppresses antibacterial T-cell responses and increases regulatory T-cell responses

**Fig 2:**
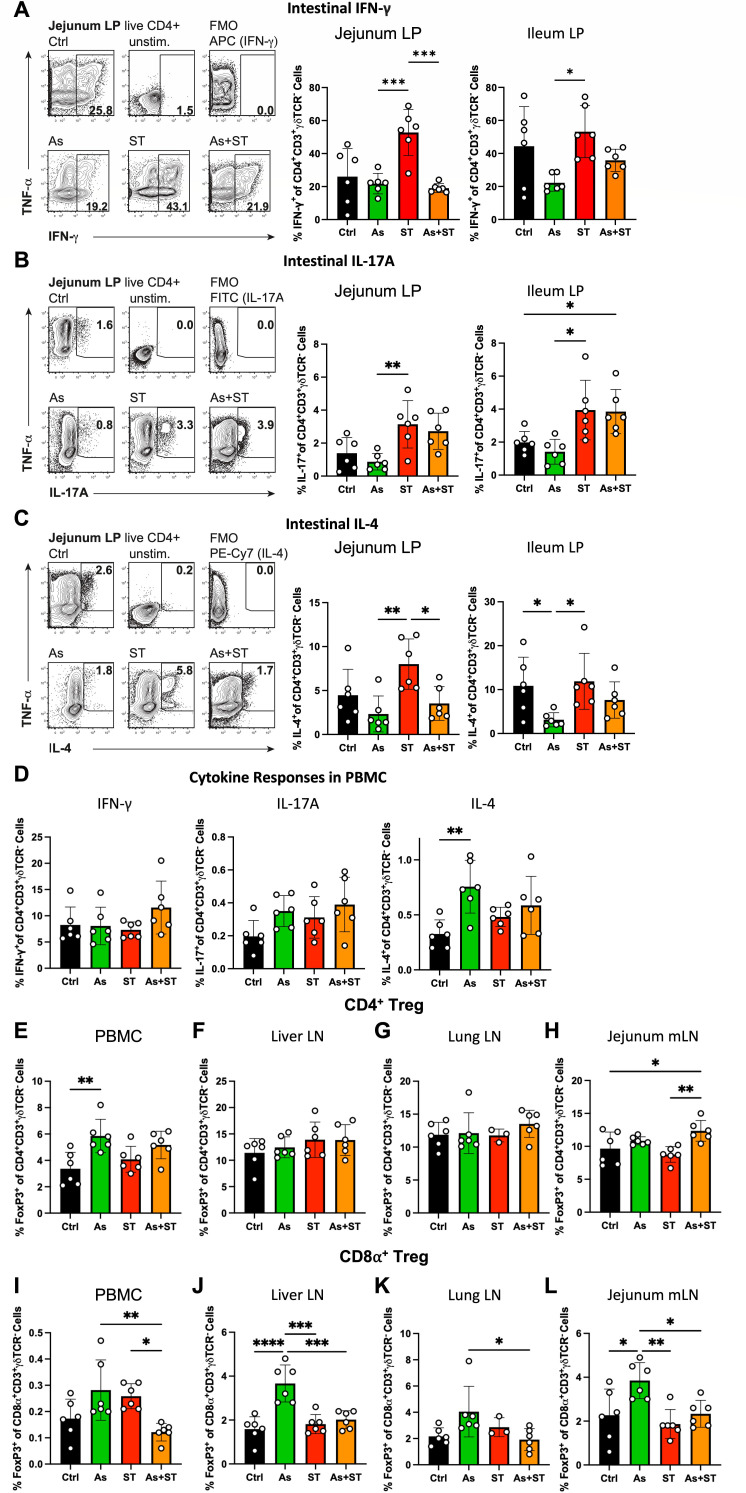
*Ascaris* infection induces a modified, regulatory type 2 T-cell response. (A) Intestinal lamina propria (LP) IFN-γ cytokine expression in T helper cells was assessed in live CD4^+^CD3^+^γδTCR^-^ cells. (B) T helper cell IL-17A expression was assessed in intestinal LP. (C) T helper cell IL-4 expression was assessed in intestinal LP. (D) Systemic T helper cytokine responses. (E) CD4^+^ Treg frequencies (FoxP3^+^CD4^+^CD3^+^γδTCR^-^) in PBMC. (F) CD4^+^ Treg frequencies in liver LN. (G) CD4^+^ Treg frequencies in lung LN. (H) CD4^+^ Treg frequencies in jejunum mLN. (I) CD8α^+^ Treg frequencies (FoxP3^+^CD4^-^CD3^+^CD8α^+^γδTCR^-^) in PBMC. (J) CD8α^+^ Treg frequencies in liver LN. (K) CD8α^+^ Treg frequencies in lung LN. (L) CD8α^+^ Treg frequencies in jejunum mLN. Statistical significance was determined by one-way analysis of variance followed by Tukey’s multiple comparisons test; * *P* ≤ 0.05; **, *P* ≤ 0.01; ***P ≤ 0.01; ****, *P* ≤ 0.001.

Type 1 and type 3 immune responses characterized by the differentiation and expansion of IFN-γ^+^ Th1 and IL-17a^+^ Th17 cells are protective against intracellular pathogens including *Salmonella* (9, 21). We therefore assessed inflammatory T-cell responses in the intestine by quantifying the frequencies of IFN-γ^+^ and IL-17A^+^ T cells via flow cytometry (Fig. 2A and B). *Salmonella* infection was associated with significantly increased IFN-γ^+^ CD4^+^ T cells compared to infection with *Ascaris* and coinfection in the jejunum lamina propria (LP) and also in the ileum LP compared to *Ascaris* infection, and these IFN-γ-producing CD4^+^ T cells were significantly dampened by coinfection with *Ascaris* (Fig. 2A). Both *Salmonella* and coinfection were associated with increased intestinal LP IL-17A^+^ T cells (trend of increased IL-17A^+^ T cells in the jejunum LP in coinfected pigs) compared to the *Ascaris* group which had the lowest proportions of IL-17A^+^ T cells (Fig. 2B). T helper cell gating is shown in the supplemental material (Fig. S3A). In contrast to *Salmonella*, helminth infections typically induce a modified, IL-4-driven type 2 immune response characterized by Th2 and Treg cells which can impair immunity to bacterial and viral infections (1, 16). We therefore assessed type 2T cell responses in the intestine by quantifying the frequencies of IL-4^+^ T cells (Fig. 2C). Interestingly, in line with previous findings (34), we did not see an indication of local Th2 responses in the intestinal LP in pigs infected with *Ascaris* or coinfected pigs (Fig. 2C), rather the *Ascaris* group had reduced frequencies of IL-4^+^ T cells compared to pigs infected with *Salmonella* (Fig. 2C).

We next assessed whether the suppression of IFN-γ^+^ T cells that we saw in the jejunum LP during coinfection could be detected systemically by assessing cytokine responses in peripheral blood mononuclear cells (PBMC; Fig. 2D). However, IFN-γ^+^ and IL-17A^+^ T cells were comparable between all groups with only a slight increase in IL-17A^+^ T cells (vs Ctrl; Fig. 2D). In contrast to intestinal IL-4 responses, both the *Ascaris* and coinfected groups showed a trend toward increased frequencies of IL-4^+^ T cells in the blood (Fig. 2D). We also assessed lung tissue and found a similar trend toward increased IL-4^+^ T cells in the *Ascaris* and coinfected groups (Fig. S4). To confirm these observations, we also assessed the expression of the Th1 transcription factor T-bet and the Th2 transcription GATA-3 in T cells in the lymph nodes of tissues impacted by *Ascaris* migration. Pigs infected with *Salmonella* had higher levels of T-bet^+^ T cells in the liver LN compared to *Ascaris* and coinfected pigs while all groups were similar in the lung LN and jejunum mLN (Fig. S5A). Notably, pigs from the *Ascaris* and coinfected groups had significantly increased GATA-3-expressing T cells compared to both the control and *Salmonella* groups in the liver LN and lung LN, while all groups were similar in the jejunum mLN (Fig. S5B). Thus, while we did not see a Th2 response in the intestine, *Ascaris* exposure resulted in a clear trend at other sites, in concordance with our previous observations where intestinal type 2 responses were detected at 28 dpi while Th2 cells were clearly seen earlier at 14 dpi in the lung (34).

To limit tissue damage, regulatory cells including Treg can suppress inflammatory responses (35). While advantageous in limiting immunopathology, Treg induced or expanded during infection can limit inflammatory responses necessary for pathogen clearance (35). Helminths, including *Ascaris*, have been shown to induce CD4^+^ Treg which can restrain anti-parasitic type 2 responses (36, 37). Therefore, we assessed for regulatory responses by quantifying FoxP3^+^ (Treg transcription factor) T cells in systemic circulation and in the lymph nodes draining the organs impacted by *Ascaris* tissue migration (Fig. 2E through H). *Ascaris-*infected pigs exhibited significantly increased CD4^+^ Treg in PBMC compared to control pigs (Fig. 2E) and the jejunum mLN coinfected pigs had significantly increased CD4^+^ Treg compared to control and *Salmonella*-infected pigs (Fig. 2H). Interestingly, no CD4^+^ Treg inductions were seen in the lymph nodes of the liver and lung (Fig. 2F and G). Treg gating and representative flow cytometry plots are shown in the supplemental material (Fig. S6).

While less frequently studied than CD4^+^ Treg, FoxP3^+^ as well CD8^+^ Treg can also be induced by various infections (35, 38). The murine helminth *Heligmosomoides polygyrus* induces robust CD8^+^ Treg capable of controlling colitis (39) and preventing autoimmune diabetes (40). We therefore quantified frequencies of CD8α^+^ Treg (Fig. 2I through L). Interestingly, while there were very low levels found in the systemic circulation for all groups, we found significantly elevated CD8α^+^ Treg in the lymph nodes of organs impacted by *Ascaris* larval tissue migration (liver, lung, jejunum) in *Ascaris*-infected pigs compared to all other groups in the liver LN and jejunum mLN and compared to coinfected pigs in lung LN (Fig. 2J through L). In contrast to CD4^+^ Treg, there was no indication of CD8α^+^ Treg induction in coinfected pigs.

Taken together, *Ascaris* infection trends toward a modified type 2 response characterized by Th2 cells as seen in the blood and lung and dominant CD4 as well as CD8α Treg cells, while pigs responded to *Salmonella* infection with relatively prominent local Th1 and Th17 cell responses. *Ascaris* infection created a modulatory immune environment wherein antibacterial T-cell responses against *Salmonella* appear attenuated.

### *Ascaris* infection modulates the host granulocyte response

**Fig 3:**
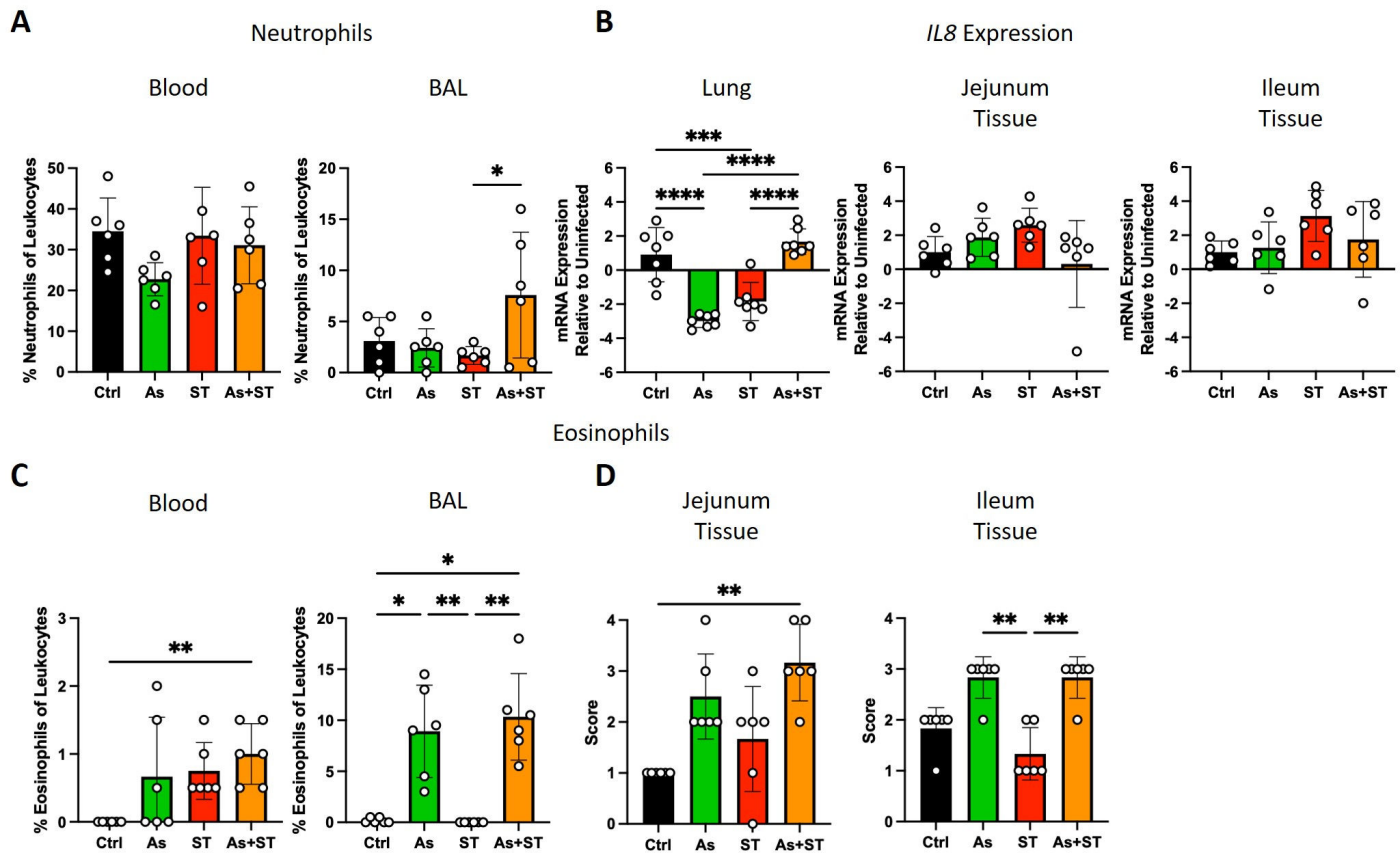
*Ascaris* infection modulates granulocyte responses. (A) Neutrophil counts in blood smears (left) and broncheoalveolar lavage (BAL) cytospins (right) as a percentage of leukocytes. (B) Bar graphs representing mean relative mRNA expression (±SD) of *IL8* in lung, jejunum, and ileum. (C) Eosinophil counts in blood smears (left) and BAL cytospins (right) as a percentage of leukocytes. (D) Tissue eosinophil counts by histological scoring of intestinal tissue as described in the methods. Data were tested for normality and statistical significance was determined by one-way analysis of variance followed by Tukey’s multiple comparisons test (Neutrophils in blood & BAL, *IL8* mRNA expression) or Kruskal-Wallis test followed by Dunn’s multiple comparisons test (all other plots); *, *P* ≤ 0.05; **, *P* ≤ 0.01; ***, *P* ≤ 0.001; ****, *P* < 0.0001.

As adaptive immune responses are a product of early innate immune signals, we turned our attention to innate immune responses. *Ascaris* and *Salmonella* differentially induce granulocytes, common first responders in different infections. Tissue-migrating *Ascaris* larvae are associated with increased levels of eosinophils which may be protective (19, 20), whereas neutrophils play a considerable role in clearing *Salmonella* (22). Therefore, we sought to quantify granulocytes in tissues impacted by the two pathogens. First, we quantified neutrophils in blood smears and bronchoalveolar lavage (BAL) cytospin preparations (Fig. 3A). Interestingly, we did not see a systemic induction of neutrophils in *Salmonella* or coinfected pigs (Fig. 3A). However, there appeared to be a decrease in mean neutrophil frequencies in the blood of *Ascaris*-infected pigs compared to all other groups (22.8% ± 3.7% vs 34.5% ± 7.5% in uninfected controls), though this difference was not statistically significant (Fig. 3A). Curiously, coinfected pigs displayed a trend toward higher levels of neutrophils in the BAL (statistically different from ST), while either pathogen alone had no influence on neutrophils in this compartment (Fig. 3A). We also assessed the ability of *Ascaris* to modulate neutrophil recruitment by assessing *IL8* mRNA levels and found that *Ascaris-* and *Salmonella-*infected pigs had decreased *IL8* mRNA expression in the lung compared to uninfected controls and coinfected pigs (Fig. 3B). We then quantified eosinophil frequencies which were very low in the blood; we did not detect eosinophils in control animals but detected low levels (≤2% of leukocytes) in the infected groups (Fig. 3C). By contrast, eosinophil frequencies in the BAL were markedly increased in both *Ascaris* and coinfected pigs compared to control and *Salmonella*-infected pigs (Fig. 3C). To further assess the impact of *Ascaris* on eosinophils, we quantified eosinophils by histology in the intestine and found that eosinophils were increased in the jejunum (vs Ctrl) of coinfected pigs and ileum (vs ST) of *Ascaris* and coinfected pigs (Fig. 3D). Thus, consistent with previous findings, *Ascaris* infection resulted in eosinophilia; hence, *Ascaris* and coinfected pigs demonstrated a type 2 bias in the innate granulocyte response which may compromise innate responses against incoming *Salmonella*.

### *Ascaris* infection decreases relative macrophage frequencies and increases M2 macrophage marker expression

**Fig 4:**
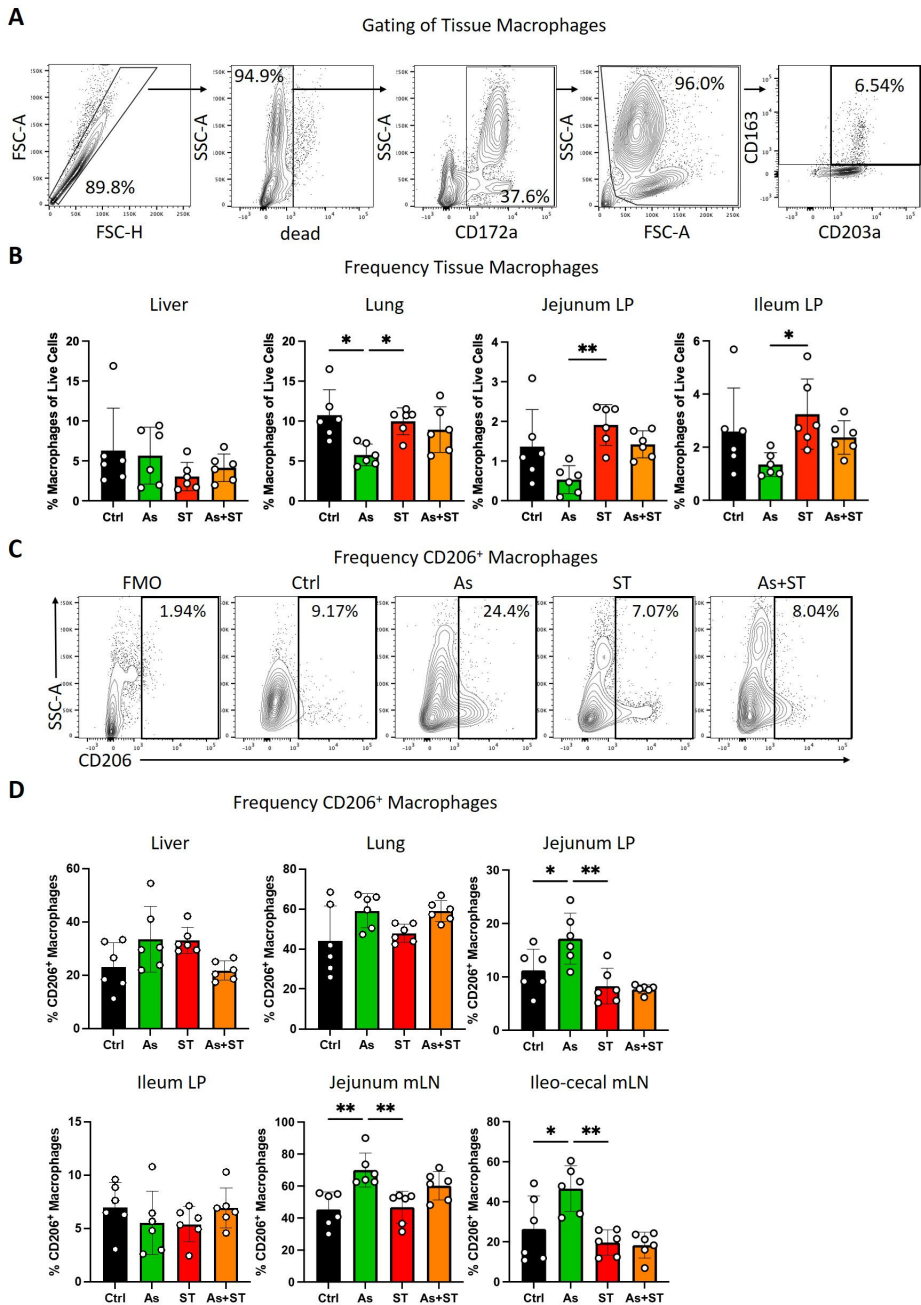
*Ascaris* infection reduces relative macrophage frequencies and induces M2 polarization. (A) Gating strategy to identify tissue macrophages, defined as live CD172a^+^CD163^+^CD203a^+^ cells. Plots report the gating of jejunum lamina propria macrophages. (B) Macrophage frequencies in liver, lung, jejunum lamina propria, and ileum lamina propria. (C) Representative flow cytometry plots of CD206^+^ macrophages in jejunum lamina propria. (D) Frequencies of CD206^+^ macrophages in different tissues. Data were tested for normality and statistical significance was determined by one-way analysis of variance followed by Tukey’s multiple comparisons test; *, *P* ≤ 0.05; **, *P* ≤ 0.01.

As macrophages are a favored site for *Salmonella* persistence (24, 26), we next assessed the impact of *Ascaris* infection on this cell type. We first assessed the frequencies of macrophages by quantifying CD172a^+^CD163^+^CD203a^+^ cells (41) in relevant tissues (Fig. 4A). Macrophage frequencies remained unchanged between groups in the liver (Fig. 4B). Interestingly, *Ascaris* infection significantly reduced relative tissue macrophage frequencies in the lung (vs Ctrl and ST), jejunum LP (vs ST), and ileum LP (vs ST; Fig. 4B). Though we were unable to assess total macrophage numbers, we confirmed that total cell numbers were not statistically different across the four groups in different organs (Table S3). In addition, we assessed frequencies of myeloid (CD172a^+^) cells in these organs and found they were comparable across all the groups (Fig. S7) while macrophage frequencies as a proportion of myeloid cells maintained similar patterns as those reported in Fig. 4 (Fig. S8).

Previous work has shown that the macrophage polarization state impacts *Salmonella* growth and persistence; M1 macrophages suppress bacterial growth while M2 macrophages are associated with proliferating bacteria (27). The mammalian mannose receptor (CD206) is expressed by several types of tissue macrophages and is considered an M2 macrophage marker as its expression is promoted by IL-4 and increased during helminth infections (41, 42). We therefore hypothesized that *Ascaris* infection would increase M2 macrophage polarization. To test this, we assessed frequencies of CD206^+^ macrophages in tissues where macrophage frequencies had been impacted by *Ascaris* infection (Fig. 4C and D). Frequencies of CD206^+^ macrophages were unchanged in the liver and ileum LP (Fig. 4D). In the lung, while not statistically significant, the frequency of CD206^+^ cells tended to increase in all infected pigs relative to uninfected controls (Fig. 4D). In the jejunum LP, *Ascaris* infection increased the frequency of CD206^+^ cells (vs Ctrl and ST; Fig. 4C and D). As *Salmonella* can persist within macrophages in gut-associated lymphoid tissue (11, 12), we also assessed the frequency of CD206^+^ macrophages in intestinal mLN. Remarkably, *Ascaris-*infected pigs had more CD206^+^ macrophages in both mLN sites (vs Ctrl and ST) indicating that macrophages at the site of *Salmonella* persistence are modulated by *Ascaris* infection. Together, these data suggest that *Ascaris* infection may reduce tissue macrophage populations while increasing M2 polarization in remaining macrophages, thereby increasing susceptibility to invading *Salmonella*.

### *Ascaris*-induced immune environment enhances macrophage susceptibility to *Salmonella*

**Fig 5:**
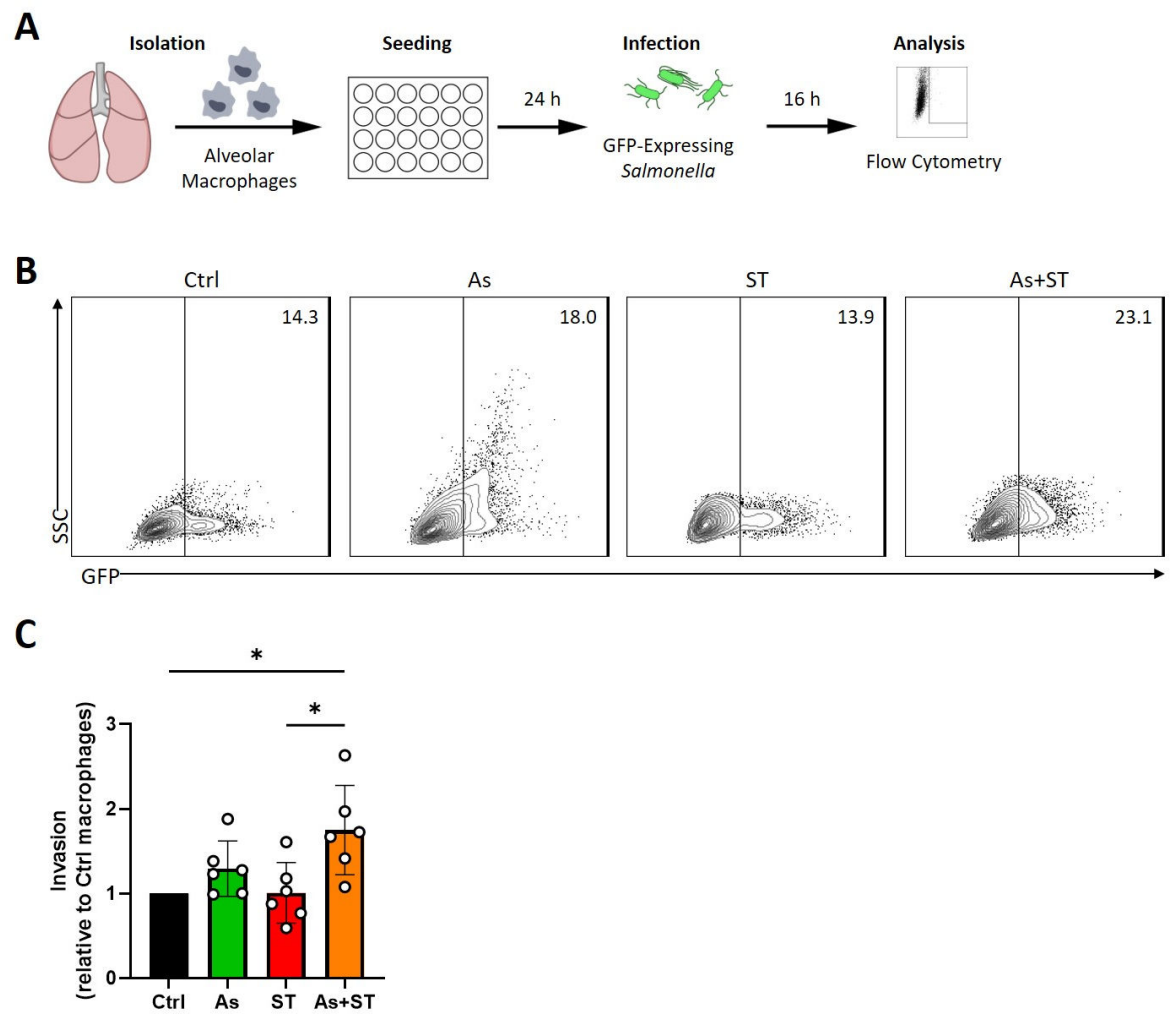
Macrophages from *Ascaris*-infected pigs are more susceptible to *ex vivo* infection with *S*. Typhimurium. (A) Experimental overview. Alveolar Macrophages were isolated from the lungs of pigs involved in this experimental infection study. Macrophages were cultured for 24 h prior to infection with GFP-expressing *Salmonella*. Sixteen hours later, cells were assessed for bacterial burden by flow cytometry. (B) Representative flow cytometry plots of GFP^+^ macrophages. (C) Columns represent mean invasion (with macrophages from Ctrl pigs set to 1.0) ± standard deviation. Statistical significance was determined by one-way analysis of variance followed by Tukey’s multiple comparisons test; *, *P* ≤ 0.05.

We next aimed to determine whether the *Ascaris-*induced immune environment had an impact on the intracellular growth of *Salmonella*. We tested this by culturing alveolar macrophages from pigs from all four groups and infecting them with GFP-expressing *Salmonella* (Fig. 5A). Then, we assessed for bacterial growth by flow cytometry. Macrophages from *Ascaris*-infected pigs had a trend toward higher intracellular *Salmonella* relative to cells from control pigs (1.29-fold increase) while macrophages from coinfected pigs had significantly more intracellular *Salmonella* (1.75-fold increase; Fig. 5B and C). We then tested whether type 2 cytokine treatments could recapitulate this finding by exposing alveolar macrophages from uninfected pigs to different cytokine treatments (Fig. S9A). Macrophages were either left untreated or treated with M1-polarizing IFN-γ, or M2-polarizing IL-4 and IL-13. Then, macrophages were infected with GFP-expressing *Salmonella* and assessed for bacterial growth by fluorescence microscopy and flow cytometry. Macrophages treated with IFN-γ showed a reduction in intracellular *Salmonella* while M2 signals increased bacterial burdens (Fig. S9B through D). Thus, pro-inflammatory signaling (IFN-γ) is protective against *Salmonella* while helminth-induced signaling (IL-4/13) impairs macrophage antibacterial activity, leading to higher intracellular bacterial burdens.

### *Ascaris* infection impairs monocyte responses and reduces relative monocyte frequencies

**Fig 6:**
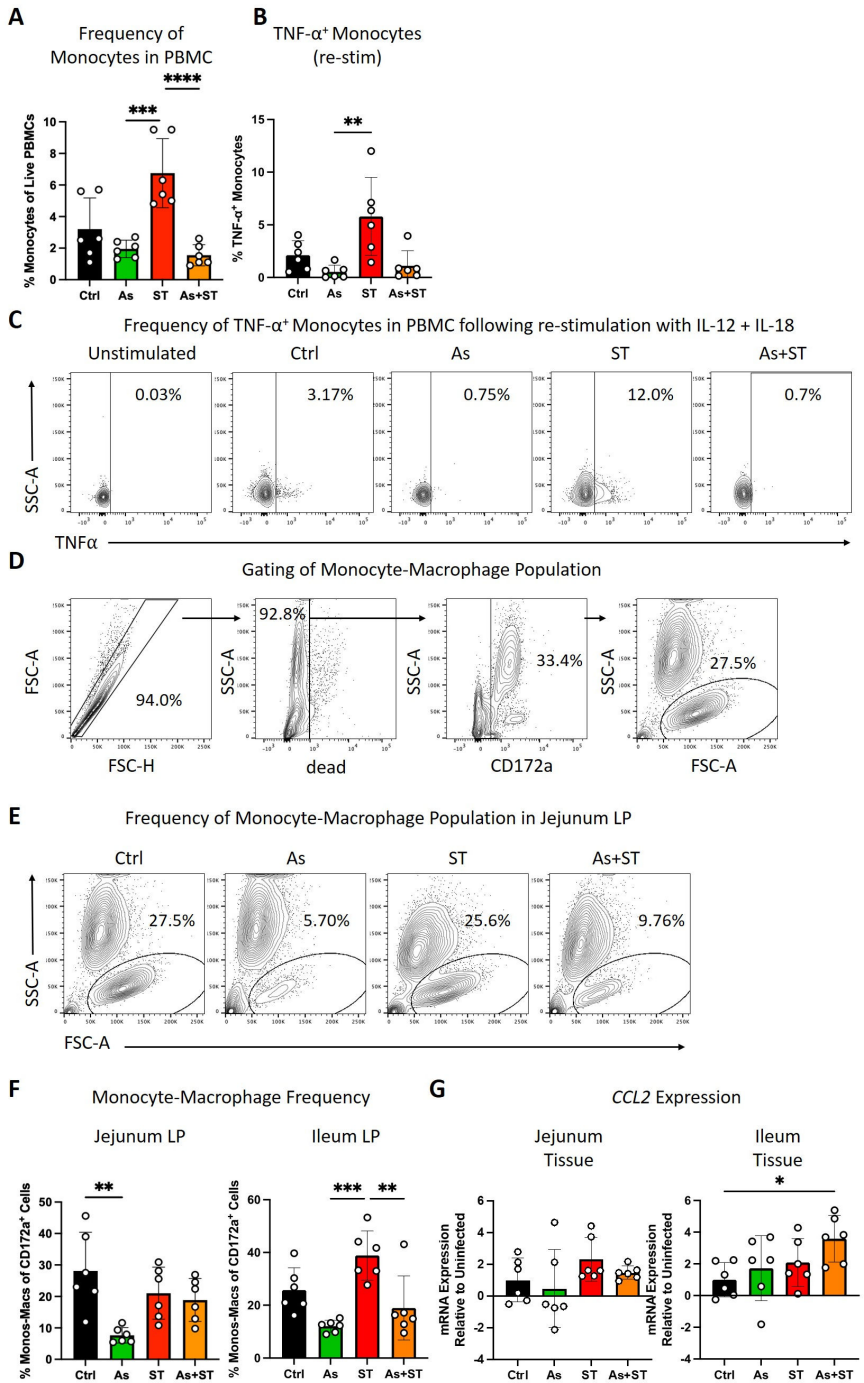
*Ascaris* infection blunts *Salmonella*-induced monocytosis and inflammatory cytokine secretion. (A) Monocyte frequencies in porcine PBMC. Monocytes were defined as live FSC^high^CD3^−^CD8^−^CD16^+^ cells. (B) Monocyte responses were assessed by TNF-α expression in monocytes stimulated with IL-12 + IL-18. (C) Representative flow cytometry plots of re-stimulated monocytes in PBMC. (D) Gating strategy to identify tissue monocytes-macrophages, defined as live CD172a^+^FSC^high^ cells. Shown here: jejunum lamina propria monocytes-macrophages. (E) Representative flow cytometry plots of monocyte-macrophage population in the jejunum lamina propria. (F) Frequencies of monocytes-macrophages in the intestine. (G) Bar graphs representing mean relative mRNA expression (±SD) of *CCL2* in the jejunum and ileum tissue. Data were tested for normality and statistical significance was determined by one-way analysis of variance followed by Tukey’s multiple comparisons test (monocyte frequencies, monocyte-macrophage frequencies, mRNA expression) or Kruskal-Wallis test followed by Dunn’s multiple comparisons test (TNF-α expression); *, *P* ≤ 0.05; **, *P* ≤ 0.01; ***, *P* ≤ 0.01; ****, *P* ≤ 0.001.

In addition to evidence for alternative activation of macrophages by *Ascaris* infection, we also observed reduced relative macrophage frequencies in the mucosal tissues of the lung, jejunum LP, and ileum LP, possibly indicating the impaired recruitment of monocytes from the blood; monocytes can be recruited to peripheral sites and differentiate into macrophages in peripheral tissues during infection (43). Furthermore, previous studies have shown that monocytes participate in *Salmonella* clearance (9, 21–23). We therefore assessed the impact of both pathogens on relative monocyte frequencies by flow cytometry. *Salmonella* infection was associated with increased relative monocyte frequencies in the blood compared to *Ascaris* and coinfected pigs (Fig. 6A). By contrast, coinfected pigs did not exhibit elevated monocytes and mean relative monocyte frequencies were lower in PBMC of *Ascaris* (2.4% ± 0.6%) and coinfected (1.6% ± 0.7%) pigs compared to uninfected controls (3.2% ± 1.9%), though not statistically significant (Fig. 6A). Next, we assessed the responsiveness of monocytes to *ex vivo* stimulation with IL-12 and IL-18. Remarkably, while monocytes from *Salmonella*-infected pigs produced TNF-α in response to cytokine stimulation (significantly different vs As), monocytes from *Ascaris* and coinfected pigs did not (Fig. 6B and C). We then sought to determine if in addition to reduced tissue macrophages and blood monocytes, the relative population of monocytes and macrophages was reduced at the site of infection in the intestine. We did so by assessing FSC^high^CD172a^+^ cells in the gut (Fig. 6E). Remarkably, *Ascaris* infection reduced the pool of monocytes and macrophages in both the jejunum LP (vs Ctrl) and the ileum LP (vs ST), while *Salmonella* increased monocyte-macrophage frequencies in the ileum LP compared to *Ascaris* and coinfected pigs (Fig. 6F). Interestingly, coinfected pigs did not display an increase in ileal LP monocyte-macrophage frequencies (Fig. 6F). Finally, we asked whether infection altered intestinal expression of monocyte chemoattractant protein-1 (MCP-1), also known as CCL2. We assessed mRNA levels of *CCL2* and did not observe a demonstrable impact of either infection on *CCL2* levels in the jejunum, while coinfected pigs had increased *CCL2* mRNA levels in the ileum compared to control pigs (Fig. 6G). Together, these data demonstrate a considerable blunting of monocyte responses against *Salmonella* by *Ascaris* and suggest an inability of the host to recruit monocytes to the site of infection to replenish monocyte and macrophage populations decreased during *Ascaris* infection. Thus, *Ascaris*-infected pigs are compromised in their response to invading *Salmonella*.

## DISCUSSION

Helminths are among the most prevalent infectious agents globally and there is ample evidence indicating increased susceptibility to co-endemic microbial pathogens in helminth-infected hosts (1, 32). Here, we show that acute *Ascaris* infection modulates host immunity rendering the host more susceptible to infection with *Salmonella*. To the best of our knowledge, this is the first study to assess and compare porcine immune responses across multiple cell types against *Salmonella* and *Ascaris* coinfections and to demonstrate a clinically meaningful interaction between the two pathogens. Our data are indicative of an *Ascaris*-modulated immune environment that is less able to deal with incoming *Salmonella*.

*Ascaris-*infected pigs demonstrated a helminth-modulated T-cell response characterized by increased IL-4 (trend) and GATA-3-producing T cells as well as Treg, while pigs infected with *Salmonella* had increased IFN-γ- and IL-17A-producing T cells compared to pigs infected with *Ascaris* (Fig. 2). Interestingly, we observed differential Treg responses, wherein classical CD4^+^ Tregs were either not induced during infection or induced in both *Ascaris* and coinfected pigs, contrasting with a rise in CD8^+^FoxP3^+^ Treg seen only in *Ascaris-*infected pigs. While CD4^+^ Tregs are known to modulate type 2 immunity (36, 37), CD8^+^ Treg are less well studied in helminth infections. Shimokawa and colleagues demonstrated that the disaccharide trehalose, produced by the murine helminth *H. polygyrus,* supported the growth of intestinal *Ruminococcus* spp. which, in turn, induced potently immunosuppressive CD8^+^ Treg in mice (40). *Ascaris* can also produce trehalose (44) and we have previously reported *Ruminococcus* to be a member of *Ascaris*’ intestinal microbiome (45). Future studies should assess the impact of acute *Ascaris* and *Salmonella* infections on intestinal microbial communities and metabolites to determine their influence on observed immune responses.

Neutrophils are early responders during *Salmonella* infection and mediate bacterial clearance (22, 46). In our study, we did not see neutrophilia in *Salmonella-*infected pigs; however, Burdick Sanchez and colleagues have shown that neutrophil levels peak in the first 24 h post-infection with *S*. Typhimurium before declining (46). Thus, by 7 dpi in our study, we would not expect to see increased levels of neutrophils. Interestingly, we found a trend toward reduced neutrophil counts during *Ascaris* infection (Fig. 3A) suggesting that incoming *Salmonella* may have fewer neutrophils to contend with in *Ascaris-*infected hosts. Consistent with prior studies (19, 20), *Ascaris* and coinfected pigs exhibited eosinophilia. During helminth infections, eosinophils support type 2 immunity by secreting IL-4, IL-5, IL-10, and IL-13 (reviewed in reference (47)). However, eosinophils can also secrete classical Th1 cytokines including IFN-γ and TNF-α (48). Nevertheless, while human eosinophils are indeed phagocytic, they are apparently less bactericidal than neutrophils (49). Our data indicate that the impact of *Ascaris* on host granulocytes renders hosts more susceptible to incoming *Salmonella,* although neutrophils eventually recover in coinfected hosts. Further studies are required to assess cytokine production and bactericidal activity of granulocytes in *Ascaris*-infected hosts.

Chen and colleagues reported monocyte recruitment to the lung and differentiation into M2-like alveolar macrophages during *Nippostrongylus brasiliensis* infection in mice (50). By contrast, our monocyte and macrophage data do not show similar tendencies in pigs based on monocyte and tissue macrophage frequencies. In agreement with Masure et al. who observed a decrease in macrophage counts in the cecum of *Ascaris*-infected pigs (20), we observed decreased relative macrophage frequencies in the jejunum and ileum during acute ascariasis (Fig. 4A). One study reported that ES from the liver fluke *Fasciola hepatica* induced apoptosis of murine peritoneal macrophages (51). Thus, further studies are warranted to determine whether *Ascaris* infection induces macrophage death. We found evidence for M2 macrophage polarization in *Ascaris*-infected pigs, macrophages in coinfected pigs are similar to those in *Salmonella*-infected pigs (Fig. 4D). However, macrophages are highly plastic (52). Nevertheless, our data from *ex vivo* infection of macrophages derived from *Ascaris* and coinfected pigs as well as *in vitro*-treated alveolar macrophages indicate that *Ascaris*-induced immune signals compromise the ability of macrophages to suppress intracellular *Salmonella* growth (Fig. 5; Fig. S9).

In a coinfection study in mice with *H. polygyrus* and *S*. Typhimurium, Rückerl et al. found that blood monocyte-derived macrophages could displace tissue-resident macrophage populations (52). We observed a drastic impairment of host monocyte responses in *Ascaris* and coinfected hosts (Fig. 6). In addition to neutrophils, monocytes are also recruited during salmonellosis and porcine monocytes can rapidly suppress intracellular *Salmonella* (23, 53). While monocytosis was observed in response to *Salmonella*, coinfected pigs did not exhibit elevated monocyte levels (Fig. 6A). Furthermore, monocytes from *Ascaris* and coinfected pigs did not produce TNF-α in response to cytokine stimulation, unlike their counterparts from *Salmonella*-infected pigs (Fig. 6B). This is consistent with previous work from our laboratory demonstrating ablated LPS-mediated TNF-α production by monocytes following treatment with *A. suum* excretory/secretory products (ES) (54). Similarly, Almeida et al. observed *A. suum* ES-mediated suppression of TNF-α responses in LPS-treated human monocyte-derived macrophages (55). Our findings from infecting BAL cells *in vitro* are also consistent with work done in other mammals. Human monocyte-derived macrophages differentiated in the presence of IL-4 had higher *Salmonella* burdens compared to macrophages differentiated in the presence of IFN-γ (56). A study of bone marrow-derived macrophages from C57BL/6 mice found that stimulating macrophages *in vitro* prior to adding *Salmonella* to the culture resulted in higher bacterial burdens compared to unstimulated macrophages or those stimulated with IFN-γ (57). Thus, acutely *Ascaris-*infected pigs may have fewer monocytes and macrophages present to fight invading pathogens, and those that are present are compromised in their ability to suppress bacterial growth.

While we observed increased *Salmonella* burdens in pigs with a concurrent *Ascaris* infection consistent with data from mice infected with *H. polygyrus* and *S*. Typhimurium, Reynolds and colleagues found that *Salmonella* coinfection was promoted by a helminth-modulated intestinal metabolome (32). Intestinal microbes not only produce bioactive metabolites but also contribute to colonization resistance to protect from various infections, including salmonellosis (58). We and others have shown that *Ascaris* infection alters the host gut microbiome (45, 59–61). Furthermore, *Ascaris* has been reported to produce acetate (62) which has been shown to enhance invasion of avian intestinal epithelial cells by *S*. Enteritidis (63). The contribution of an *Ascaris-*modulated microbiome and metabolome to susceptibility to microbial infection is an exciting area for further research.

Limitations of this study arose from the study design and technical limitations of working with pigs compared to studies in mice. Assessing bacterial burdens at 7 dpi only allows an assessment of the pathogenesis of infection between acute salmonellosis which peaks around 2–3 dpi (64) and persistence. Extended studies are required to assess bacterial burdens weeks and months after infection. However, assessing *Salmonella* burdens at 7 dpi provides an opportunity to assess host lymphoid tissue colonization and persistence in a short experiment setting. Similarly, we have likely missed the expected neutrophilia during salmonellosis due to the experimental timeline. Due to the size of porcine organs, we were limited to an assessment of frequencies rather than total target cell counts as are commonly reported in studies using mice. Though we did not see any differences in frequencies of myeloid cells across the four groups (Fig. S7), we were only able to report relative changes in frequencies of macrophages and monocytes; therefore, it is possible that reduced frequencies of macrophage and monocyte-macrophage populations reported here are indeed only relative decreases due to increased levels of eosinophils (Fig. 3). An intriguing question not answered by our study design is whether *Salmonella* can modulate host responses against *Ascaris*. A previous study by Steenhard et al. in which pigs were first infected with *S*. Typhimurium, then infected with 2,500 *A*. *suum* eggs twice weekly for 7 weeks found no impact on *Salmonella* burdens when *Ascaris* was introduced starting at 3 dpi and the authors reported no impact of *Salmonella* on worm burdens (65). Importantly, immune parameters were not studied in detail in this study. Neutrophils and macrophages are modulated by *Salmonella*, allowing intracellular survival (27, 66). *Salmonella*-infected pigs also exhibit a disrupted intestinal microbiome (67, 68). Thus, whether *Salmonella* might influence ascariasis directly through its interaction with immune cells or indirectly *via* the microbiome is worthy of further study.

**Fig 7:**
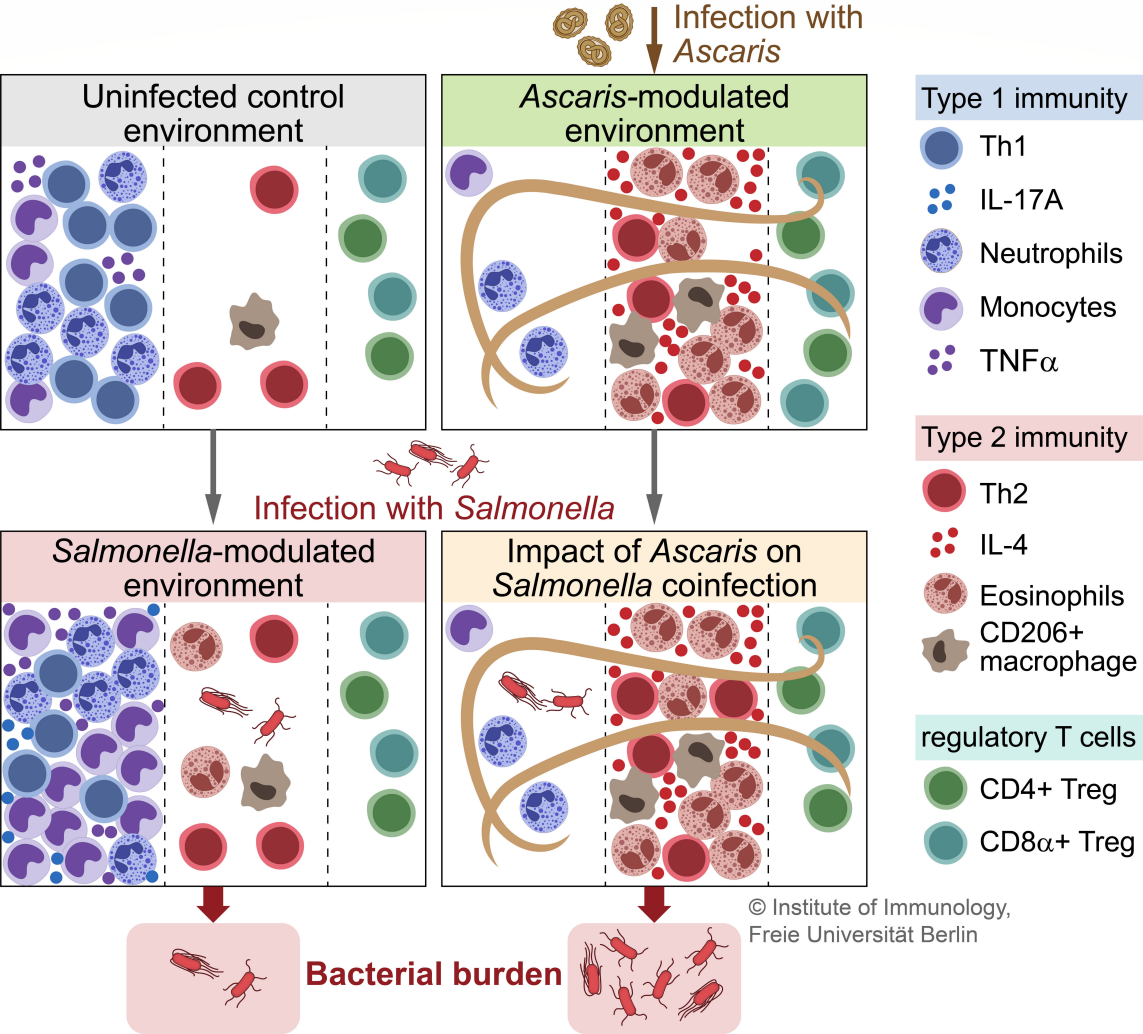
*Ascaris* infection modulates host immunity creating an immune environment more permissive for invading *Salmonella* (graphical abstract). *Ascaris* and *Salmonella* induce opposing immune responses. During an ongoing *Ascaris* infection, invading *Salmonella* encounters fewer neutrophils, monocytes, and macrophages. The remaining monocytes and macrophages are less responsive to *Salmonella* and exist within a type 2 immune environment (more IL-4, less pro-inflammatory cytokines). Collectively, an *Ascaris-*modulated immune environment allows *Salmonella* to establish itself, leading to higher bacterial burdens in coinfected pigs.

Collectively, our observations demonstrate that *Ascaris* is capable of immunomodulation even during acute infection. This modulation establishes an immune environment permissive to infection with other pathogens (summarized in Fig. 7). Our results in a clinically relevant model suggest that helminth infections should be more closely monitored during pig production. Further studies on other coinfecting pathogens of interest, such as *Campylobacter jejuni, Streptococcus suis,* influenza, and porcine reproductive and respiratory syndrome viruses are warranted.

## MATERIALS AND METHODS

### Experimental infection of pigs

See Text S1 for the experimental infection protocol.

### Necropsy and tissue sampling

Pigs were sedated using ketamine hydrochloride (33 mg/kg, Ursotamin, Serumwerk Bernburg AG, Bernburg, Germany), xylazine (6 mg/kg, Xzlavet, CP-Pharma GmbH, Burgdorf, Germany), and azaperone (4 mg/kg, Stresnil, Janssen-Cilag GmbH, Neuss, Germany) and euthanized by intracardial injection of T61 (10 mg/kg of tetracaine hydrochloride, mebezonium iodide, and embutramide, Intervet Deutschland GmbH, Unterschleißheim, Germany). See Text S1 for tissue sampling protocols.

### *Salmonella* burden determination

*Salmonella* burden was determined in cell suspensions obtained from tissue homogenates at different sites (jejunal mLN, ileocecal mLN, and spleen). Cell suspensions were collected and enumerated using the CASY immediately following tissue homogenization and 10^7^ host cells were plated onto LB plates in replicate (five plates per sample) supplemented with 50 µg/mL nalidixic acid (Carl Roth GmbH) and incubated overnight at 37°C. The next day *S*. Typhimurium colonies were counted.

### Leukocyte isolation and differential leukocyte counts

See Text S1 for leukocyte isolation and counting methods.

### Cell stimulation and flow cytometry

See Text S1 for cell stimulation and flow cytometry methods.

### Histological analysis

Formalin-fixed tissues were embedded in paraffin, cut into sections using a microtome, and stained with hematoxylin and eosin for histological analysis. See Text S1 for histological scoring.

### Gene expression analysis

Frozen tissue samples were processed for RT-qPCR analysis using the InnuPrep RNA Mini Kit (Analytik Jena, Jena, Germany) according to the manufacturer’s instructions (see Text S1).

### *Salmonella* infection assays

See Text S1 for *in vitro Salmonella* infection assays.

### Statistical analysis

Statistical analysis and visualization of data were performed with GraphPad Prism software (version 9, Dotmatics, Boston, MA, USA) as described in Text S1.

## SUPPLEMENTAL MATERIAL

Supplemental Material msphere.00478-24-s0001.pdf Fig. S1 to S9, Tables S1 to S3, and Text S1.

ASM does not own the copyrights to Supplemental Material that may be linked to, or accessed through, an article. The authors have granted ASM a non-exclusive, world-wide license to publish the Supplemental Material files. Please contact the corresponding author directly for reuse.
