# Supplementary material for: Concurrent Ascaris infection modulates host immunity resulting in impaired control of Salmonella infection in pigs
Source: mSphere. 2024 Aug 14;9(9):e00478-24. doi: 10.1128/msphere.00478-24 (PMC11423588; doi:10.1128/msphere.00478-24)
Supplement: Supplemental Material — Fig. S1 to S9, Tables S1 to S3, and Text S1. [file msphere.00478-24-s0001.pdf]

Supplemental Material

Concurrent *Ascaris* infection modulates host immunity resulting in impaired control of *Salmonella* infection in pigs

Ankur Midha[a#], Larissa Oser[a], Josephine Schlosser-Brandenburg[a], Alexandra Laubschat[a], Robert M. Mugo[a], Zaneta D. Musimbi[a], Philipp Höfler[a], Arkadi Kundik[a], Rima Hayani[a], Joshua Adjah[a], Saskia Groenhagen[a], Malte Tieke[a], Luis E. Elizalde-Velázquez[a], Anja A. Kühl[b], Robert Klopfleisch[c], Karsten Tedin[d], Sebastian Rausch[a], Susanne Hartmann[a]

[a]Institute of Immunology, Centre for Infection Medicine, Department of Veterinary Medicine, Freie Universität Berlin, Berlin, Germany

[b]Charité Universitätsmedizin Berlin, corporate member of Freie Universität Berlin and Humboldt-Universität zu Berlin, iPATH.Berlin, core unit of Charité, Campus Benjamin Franklin, Berlin, Germany

[c]Institute of Veterinary Pathology, Department of Veterinary Medicine, Freie Universität Berlin, Berlin, Germany

[d]Institute of Microbiology and Epizootics, Centre for Infection Medicine, Department of Veterinary Medicine, Freie Universität Berlin, Berlin, Germany

#Address Correspondence to: Ankur Midha, ankur.midha@fu-berlin.de

Supplemental Material

Text S1. Supplemental materials and methods.

Experimental infection of pigs

A total of 24 weaning hybrid (German landrace and large white) pigs of both sexes were obtained from a conventional breeder (Brandenburg, Germany) at 6 weeks of age. The pigs were allowed to acclimate for 11 days prior to the experiment. Water was given ad libitum and food was provided twice daily according to weekly body weight measurements. Wood chips were used as bedding and straw and toys were provided for enrichment. The pigs were randomly assigned to four groups, balanced for body weight and sex. The four groups included uninfected controls (Ctrl, n=6), pigs infected with *A. suum* (As, n=6), pigs infected with *S.* Typhimurium (ST, n=6), and animals infected with both pathogens (As+ST, n=6).

Infective *A. suum* eggs were collected and prepared as previously described (1). Adult female worms obtained from a local slaughterhouse were cultured overnight at 37 °C. Eggs released into the culture medium were collected, washed, and incubated at room temperature in the dark for 8 weeks. Pigs were orally infected with 2000 embryonated *A. suum* eggs/day for 4 days. The inoculum was fed to the pigs on store-bought waffles.

S. Typhimurium definitive type 104 (strain BB440) is a nalidixic acid resistant zoonotic pathogen and was used because it was originally obtained from a pig with acute salmonellosis (2). *Salmonella* were grown in Luria-Bertani (LB) broth (Carl Roth GmbH, Karlsruhe, Germany) with aeration at 37 °C to an optical density at 600 nm of 1.5-2.0. Pigs were orally inoculated with 10^7 colony forming units (CFU) delivered on waffles, as for the *A. suum* inoculum. Pigs were closely monitored (body weight, visual observation of pigs and their feces) in the days following infection.

Dissection timepoints were 14 dpi for *A. suum* and 7 dpi for *S.* Typhimurium. The same time points were maintained for coinfected animals and uninfected controls were included at each timepoint. The

experimental timeline was staggered across 3 separate dissection days to accommodate the large volume of sample processing.

Tissue Sampling

The trachea was clamped and the lungs were removed. Then, the right lung was flushed with 200 mL of phosphate-buffered saline (PBS) supplemented with 2 mM ethylenediaminetetraacetic acid (EDTA) to obtain broncheo-alveolar lavage (BAL) cells. BAL samples were filtered through a 70 µm cell strainer and stored on ice for further processing. Tissue samples for cellular phenotyping from the lung, liver, spleen, jejunum LP, ileum LP, and mesenteric lymph nodes (mLN) were collected and pooled in wash medium (Roswell Park Memorial Institute 1640 medium with 1% fetal calf serum (FCS), 100 U/mL penicillin, 100 µg/mL streptomycin; all PAN-Biotech GmbH, Aidenbach, Germany) and stored on ice for further processing. One tissue sample (2 x 2 cm) from each of three different regions (three total per organ) were collected for lung, liver, and spleen. For jejunum LP, a 10 x 10 cm tissue sample was collected mid-way through the jejunum. For ileum LP, a 10 x 10 cm tissue sample free of Peyer's patches was collected ~20-30 cm proximal to the cecum. For lung LN samples, five LN were collected. For liver LN, two LN were collected, and for intestinal mLN samples, 10 LN were collected.

Tissue samples for histological analysis were stored in formalin (Roti®-Histofix 10%, Carl Roth GmbH) overnight at room temperature before being transferred to fresh formalin and stored at 4 °C until further processing. Tissue samples for gene expression analysis were snap frozen in liquid nitrogen before being transferred for long term storage at -80 °C.

Leukocyte Isolation

Peripheral blood mononuclear cells (PBMC) were isolated from blood collected by heart puncture in EDTA coated tubes (S-Monovette®, Sarstedt AG & Co. KG, Nümbrecht, Germany). Blood was diluted 1:2 in 0.9% NaCl and subjected to density gradient centrifugation using Pancoll human solution (density 1.077 g/mL, PAN-Biotech). Splenic and lymph node leukocytes were isolated by mechanical disruption of tissue samples and passage through a 70 µm cell strainer. Lung and liver leukocytes were isolated

from multiple tissue pieces which were mechanical homogenized, pooled, and pre-digested using the Lung and Liver Dissociation Kits for mice and the gentleMACS™ Dissociator (Milteny Biotec, Bergisch Gladbach, Germany) using the manufacturer's protocol with 3X enzyme concentrations.). Small intestinal lamina propria leukocytes were isolated from tissue samples of approximately 10 cm length from the jejunum and ileum after removal of fat, muscle, and connective tissue. Tissues were homogenized and digested with 5 mg/mL Liberase TM, 5 mg/mL Liberase DH, and 4 mg/mL DNaseI (All Roche Diagnostics GmbH, Mannheim, Germany) at 37 °C under gentle agitation (2 x 20 min) prior to mechanical disruption by passage through a 190 µm mesh. All cell suspensions were washed with wash medium and treated with erythrocyte lysis buffer (0.01 M $KHCO_3$, 0.155 M NH_4Cl, 0.1 mM EDTA, pH 7.5) before being resuspended in complete culture medium (cIMDM; Iscove's Modified Dulbecco's Medium supplemented with 10% FCS, 100 U/mL penicillin, 100 µg/mL streptomycin, all PAN-Biotech). Cells were counted using the Cell Counter & Analyzer CASY (OMNI Life Science GmbH & Co. KG, Bremen, Germany) and cell suspensions adjusted to 2 x 10^7 cells/mL for further use.

Differential Leukocyte Counts

Leukocyte counts were performed on blood smears and cytospins of BAL fluid cells. Cells on microscope slides were fixed using ethanol and propanol (ROTI®Fix spray, Carl Roth GmbH) then Romanowsky stained (DiffQuick, Labor + Technik, Eberhard Lehmann GmbH, Berlin, Germany). 200 leukocytes were counted and classified to determine percentages of lymphocytes, neutrophils, eosinophils, basophils, and macrophages/monocytes.

Cell Stimulation and Flow Cytometry

To assess cellular markers, cells were plated at 2 x 10^6 cells per well in conical 96 well plates, blocked with mouse serum (1:500), and stained for surface and intracellular markers using the antibodies listed in Table S1 for flow cytometry analysis (BD FACS Canto II, BD FACSARIA III, Flowjo version 10, all BD Life Sciences, Franklin Lakes, NJ, USA) following standard protocols (3). Intranuclear and intracellular

marker were stained after fixation and permeabilization of cells with the FoxP3/Transcription Factor staining buffer set (Thermo Fisher) or Cytofix/Cytoperm (BD Biosciences), respectively.

For cytokine expression analysis of T cells, cells were plated at 3 x 10^6 per well in a round bottom 96 well plate and allowed to rest overnight at 37°C for T cell cytokine staining. Cells were then stimulated with Phorbol myristate acetate (PMA; 50 ng/mL, Sigma-Aldrich) plus ionomycin (500 ng/mL, Sigma-Aldrich) for 3.5 h in presence of Brefeldin A (1 µg/mL, eBioscience) during the last 3h of restimulation.

For cytokine expression analysis of monocytes, cells were plated at 1 x 10^6 cells per well in round bottom 96 well plates and stimulated with 100 ng/mL recombinant porcine IL-12p70 and 100 ng/mL recombinant porcine IL-18 (both from R&D Systems; Minneapolis, MN, USA) for 13 h at 37 °C and 5% CO_2 in the presence of Brefeldin A (3 µg/mL; ThermoFisher; Waltham, MA, USA) for the last 10 h of stimulation.

Histological Scoring

For liver tissue scoring was performed according to Con A-induced hepatitis scoring involving the summation of scores for lobular and portal inflammation and necrosis (4). For lung tissue scoring was done according to BCG infection scoring (5). For jejunum and ileum, scoring was carried out according to small intestinal inflammation scoring from Erbet et al. (6). For tissue eosinophil counts, scoring was as follows: 0, normal (physiologic conditions); 1, minimally increased; 2, mildly increased; 3, moderately increased; 4, markedly increased.

Gene Expression Analysis

Frozen tissue samples were processed for RT-qPCR analysis using the InnuPrep RNA Mini Kit (Analytik Jena, Jena, Germany) according to manufacturer's instructions. Extracted RNA was transcribed to cDNA using the High Capacity RNA-to-cDNA kit from Applied Biosystem (ThermoFisher; Waltham, MA, USA). Amplification and detection were performed in 96-well optical plates with SYBR-green (Both Applied Biosystems, ThermoFisher). Amplifications were performed in duplicate in a final volume of 20 µL containing 10 µL of 2X SYBR Green I Master Mix and 5 µM of each primer (Table S2). mRNA

expression was normalized to the housekeeping gene glycerinaldehyd-3-phosphat-dehydrogenase (*GAPDH*) and standard curves were generated to calculate the efficacy of each primer pair. Relative expression was calculated using the $2^{-\Delta\Delta CT}$ method (7).

***Ex Vivo Salmonella* Infection Assay With Cells from Experimental Infection**BAL cells from all four groups were plated at approximately 3 x 10^5 cells per well in 24-well cell culture plates and incubated for 1 h at 37 °C. After 1 h, the cells were washed and replaced with fresh cIMDM, leaving the alveolar macrophages attached to the plate which were incubated overnight. The next day, cells were infected with *S.* Typhimurium (ATCC 14028 gyrA (D87Y); strain 8642/KT8640 (pFPV25.1)) grown in aeration at 37 °C at a multiplicity of infection of 10 based on the number of BAL cells plated (3 x 10^6 bacterial cells added/well). After addition of bacteria to cell cultures, plates were centrifuged at 150 x *g* for 10 minutes and incubated at 37 °C for 50 mins followed by a change of cell culture medium to cIMDM supplemented with gentamicin 50 µg /mL (PAN-Biotech) and further incubation for 1 h. Medium was then changed to cIMDM with gentamicin 10 µg /mL and incubated overnight (total infection time ~ 16 h). The next day, cells were washed with PBS and removed from the cell culture plates using trypsin-EDTA (PAN-Biotech) prior to staining with fixed viability dye and analysis by flow cytometry.

***In Vitro Salmonella* Infection Assay with Cytokine Treatments**

BAL cells from uninfected pigs were plated, washed, and rested as mentioned in the previous section. The next day, cells were either left untreated, treated with 100 µg/mL IFN-γ, or 10 µg/mL of both IL-4 and IL-13 (IFN-γ and IL-4, R & D Systems; IL-13, Kingfisher Biotech, Saint Paul, MN, USA). Two days later cells were infected with *S.* Typhimurium (ATCC 14028 gyrA (D87Y); strain 8642/KT8640 (pFPV25.1)) grown in aeration at 37 °C at a multiplicity of infection of 10 based on the number of BAL cells plated (3 x 10^6 bacterial cells added/well). After addition of bacteria to cell cultures, plates were centrifuged at 150 x *g* for 10 minutes and incubated at 37 °C for 50 mins followed by a change of cell culture medium to cIMDM supplemented with gentamicin 50 µg /mL (PAN-Biotech) and further incubation for 1 h. Medium was then changed to cIMDM with gentamicin 10 µg /mL and incubated overnight (total infection time ~ 16 h). The next day, cells were analyzed by microscopy and imaged on an Axiovert.A1

microscope equipped with a Colibri 7 solid-state light source and Axiocam 503 mono microscope camera using Zen (v 2.3) software (Carl Zeiss, Oberkochen, Germany). Cells were then washed with PBS and removed from the cell culture plates using trypsin-EDTA (PAN-Biotech) prior to staining with fixed viability dye and analysis by flow cytometry.

Statistical Analysis

Statistical analysis and visualization of data were performed with GraphPad Prism software (version 9, Dotmatics, Boston, MA, USA). Data were tested for normality using the Shaprio-Wilk test. Parametric data were analyzed by one-way analysis of variance followed by Tukey's multiple comparisons test. Non-parametric data were analyzed by the Kruskal-Wallis test followed by Dunn's multiple comparisons test. Differences were considered statistically significant at $p < 0.05$ and indicated as follows: $p \leq 0.05$ (*), $p \leq 0.01$ (**), $p \leq 0.01$ (***), and $p \leq 0.001$ (****).

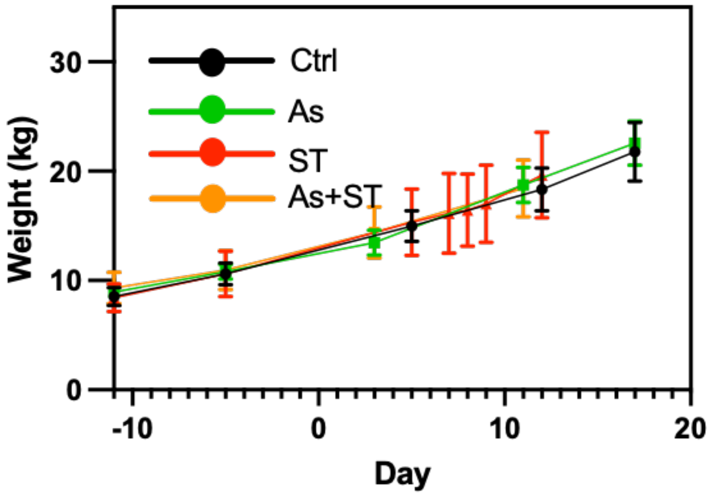

Figure S1. Infections had no impact on pig weight gain. Shown are average weights (kg) ± SD across groups throughout the experiment.

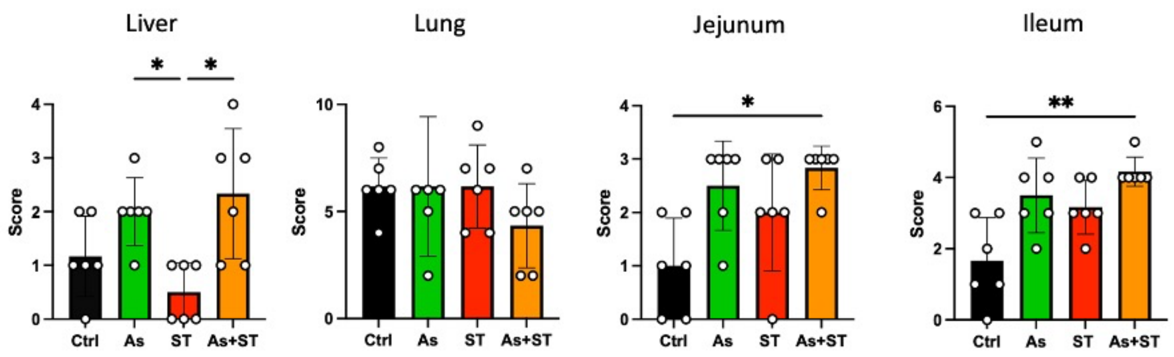

Figure S2. Histopathology scoring of tissues impacted by *Ascaris* and *Salmonella*. Pathology scores determined as described in the methods section. Statistical significance was determined by one-way analysis of variance followed by Tukey's multiple comparisons test, * $p \leq 0.05$, ** $p \leq 0.01$.

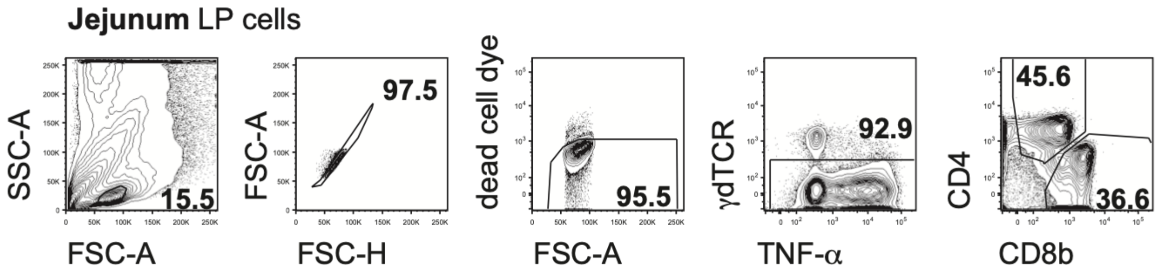

Figure S3. Gating strategy to identify T helper cells as CD3$^+$CD4$^+$CD8$^-$γδTCR$^-$ cells. Shown here: Jejunum LP T helper cells.

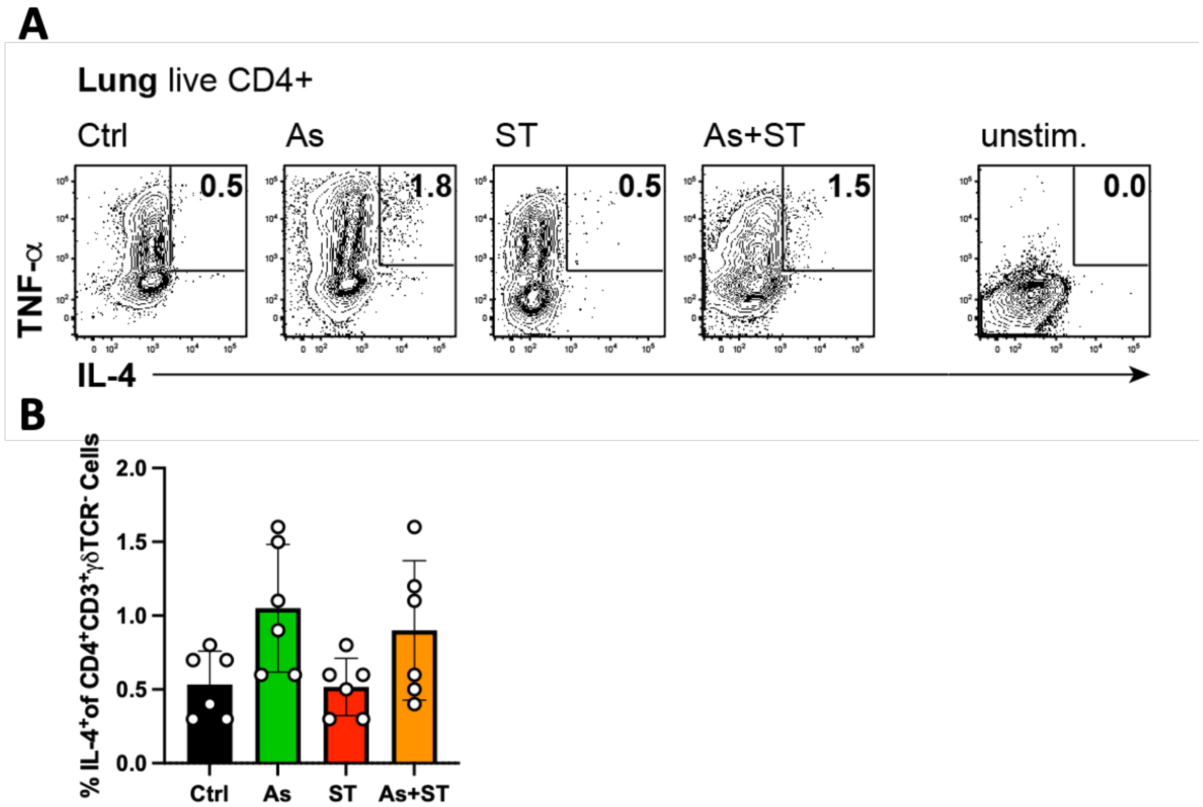

Figure S4. T helper cell IL-4 expression in lung tissue. **A.** Representative flow cytometry plots of IL-4$^+$ T helper cells. **B**. Frequencies of IL-4$^+$ T helper cells.

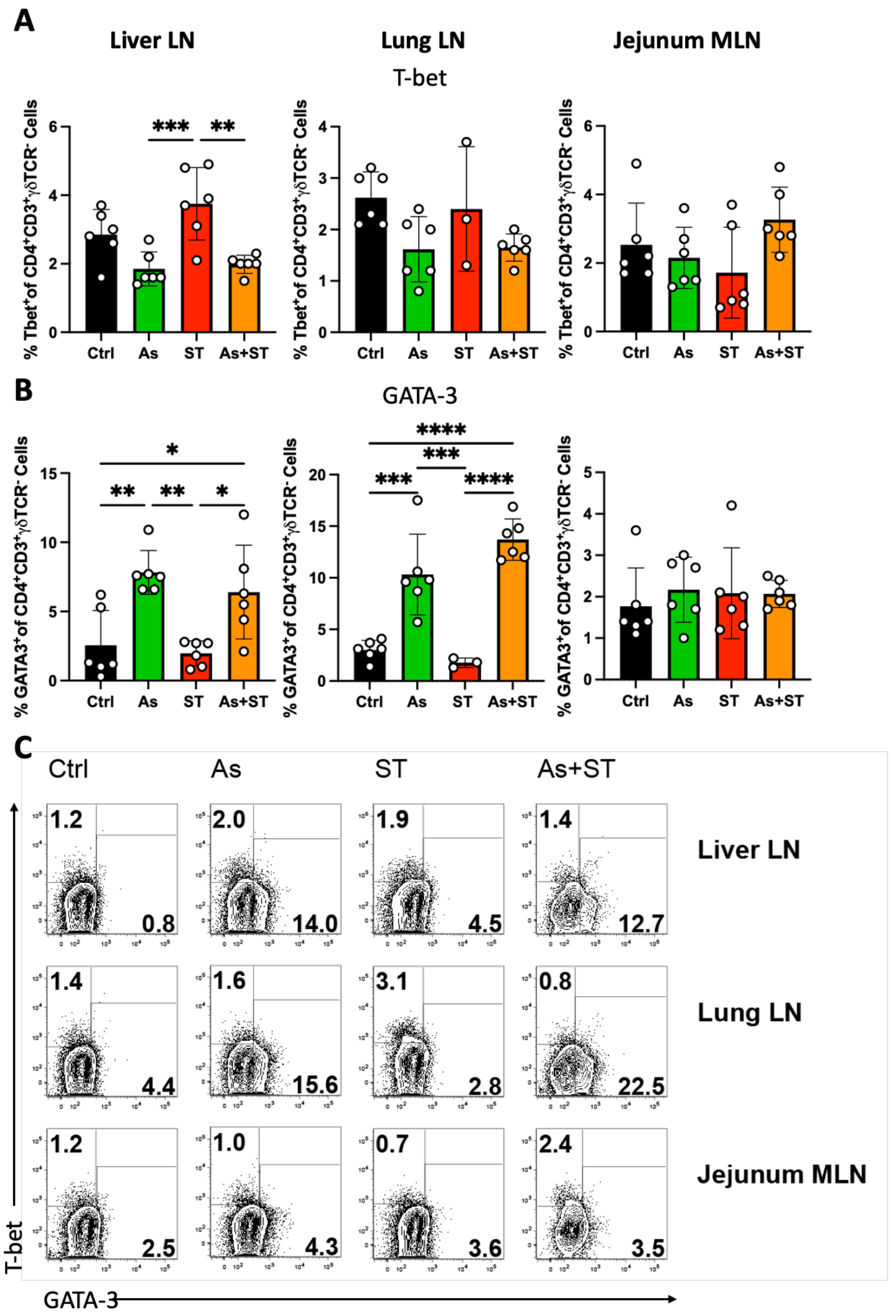

Figure S5. Transcription factor expression by T helper cells in the lymph nodes of tissues impacted by *Ascaris* larval migration. **A.** Frequencies of T-bet⁺ T helper cells in the lymph nodes (LN) of liver, lung, and jejunum. **B.** Frequencies of GATA-3⁺ T helper cells in liver LN, lung LN, and jejunum MLN. **C.** Representative flow cytometry plots of transcription factor-expressing T helper cells.

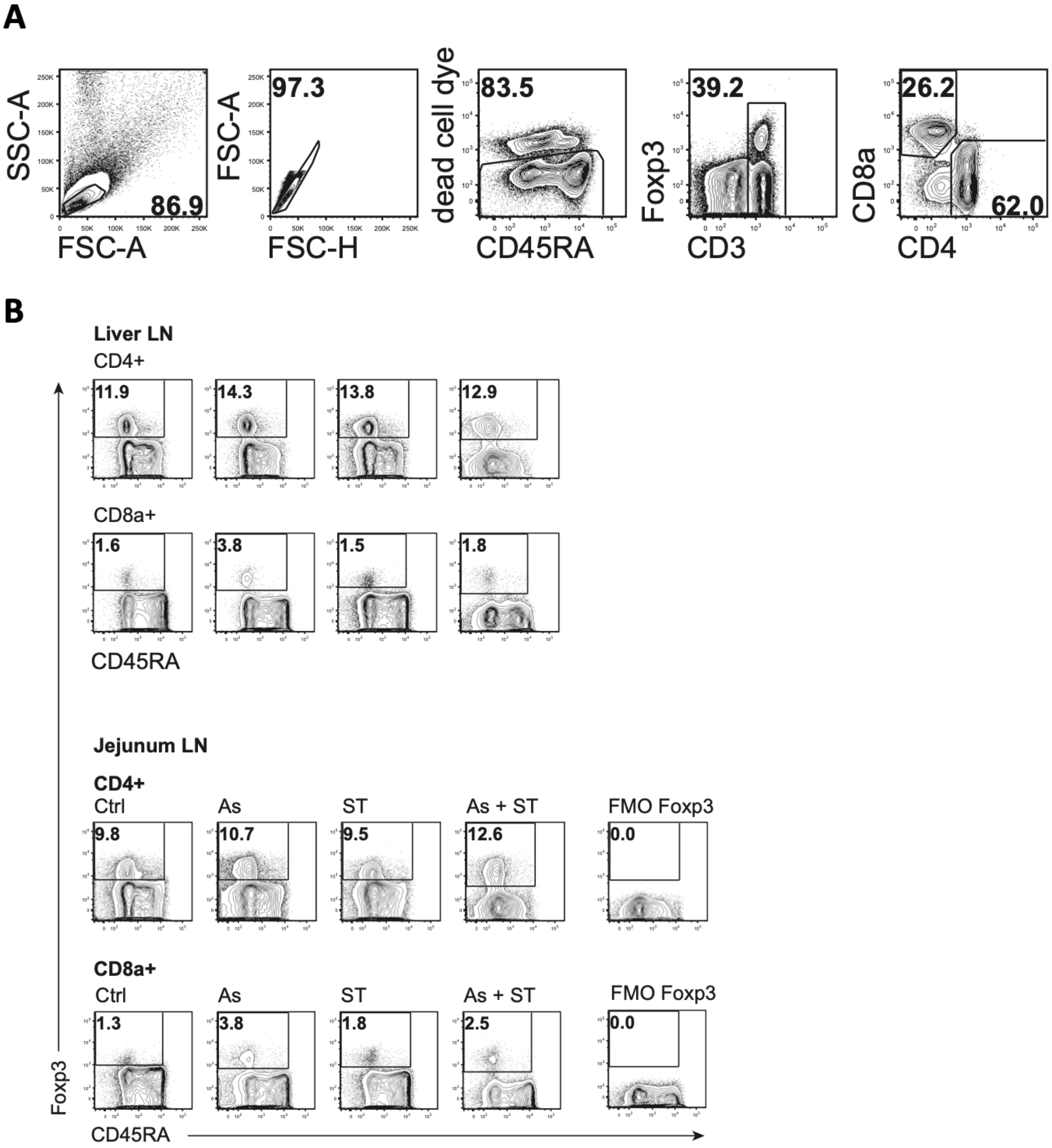

Figure S6. A. Gating strategy to identify $CD4^+$ and $CD8\alpha^+$ regulatory T cells. Shown here: liver LN **B.** Representative flow cytometry plots of regulatory T cells in the lymph nodes of liver and jejunum.

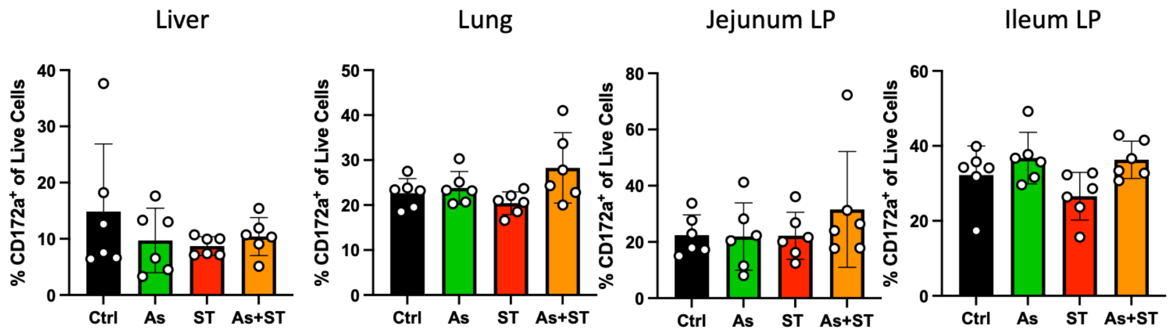

Figure S7. Myeloid cell frequencies in liver, lung, jejunum lamina propria (LP), and ileum LP. Myeloid cells defined as live $CD172a^{+}$ cells. Data were tested for normality and statistical significance was determined by one-way analysis of variance (liver, lung, ileum LP) or by Kruskal-Wallis test (Ileum LP).

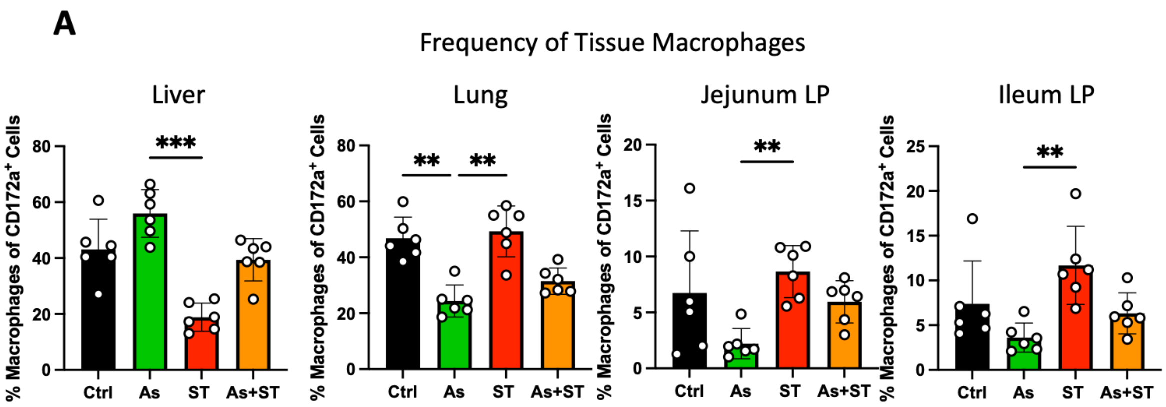

Figure S8. A. Frequencies of tissue macrophages (live CD172a$^+$CD163$^+$CD203a$^+$ cells) as a proportion of myeloid (CD172a$^+$) cells. Data were tested for normality and statistical significance was determined by one-way analysis of variance followed by Tukey's multiple comparisons test (total macrophage counts in liver), or by Kruskal-Wallis test followed by Dunn's multiple comparisons test (all others), * $p \leq 0.05$, ** $p \leq 0.01$, *** $p \leq 0.001$.

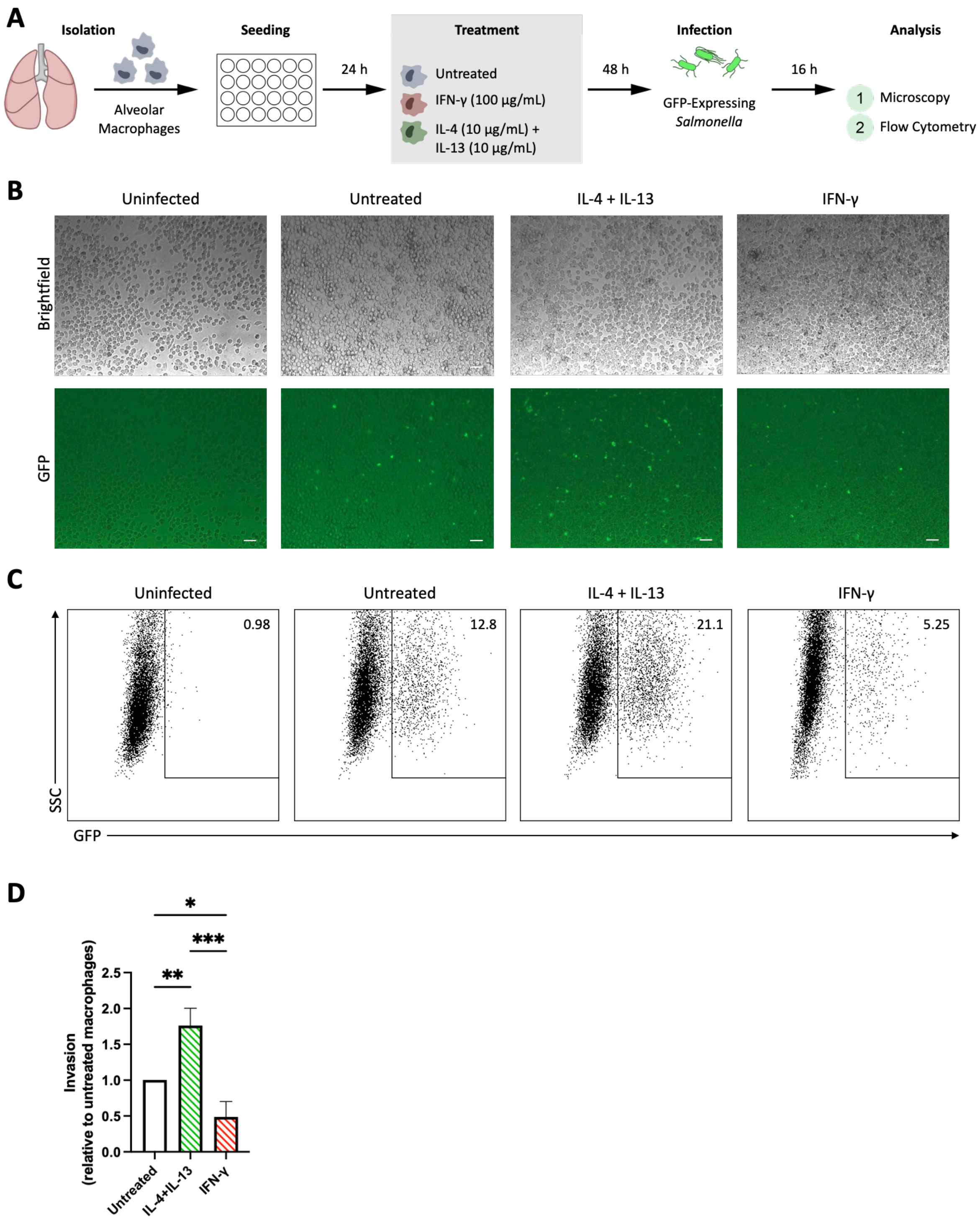

Figure S9. *Ascaris*-modulated immune environment enhances macrophage susceptibility to *Salmonella* infection. **A.** Experimental overview. Alveolar macrophages were isolated from the lungs of uninfected pigs. Macrophages were cultured in culture media with and without cytokine treatments for 48 h prior to infection. Macrophages were infected at a multiplicity of infection of 10 with GFP-expressing *Salmonella* Typhimurium. Sixteen hours later, cells were assessed for bacterial burden by fluorescence

microscopy and flow cytometry. **B.** Representative images of GFP-expressing *Salmonella* infection of macrophages. Cells visualized with 20x objective. Data are representative of three independent experiments. **C.** Representative flow cytometry plots of GFP^+ macrophages. Data are representative of three independent experiments. **D.** Columns represent mean invasion (with untreated macrophages set to 1.0) from three independent experiments + standard deviation. Statistical significance was determined by one-way analysis of variance followed by Tukey's multiple comparisons test, * $p \leq 0.05$, ** $p \leq 0.01$, *** $p \leq 0.01$.

Table S1. Antibodies used in this study

Target	Clone	Reactive Species	Host Species	Isotype	Conjugate	Manufacturer	Product Code
CD3e	BB23-8E6	Pig	Mouse	IgG2b	Biotin	Southern Biotech	SBA-4511-08
CD4a	74-12-4	Pig	Mouse	IgG2b	PerCP-Cy5.5	BD Biosciences	561474
CD8a	76-2-11	Pig	Mouse	IgG2a	-	ThermoFisher	MA5-28717
CD8a	76-2-11	Pig	Mouse	IgG2a	FITC	ThermoFisher	MA5-28714
CD8b	PPT23	Pig	Mouse	IgG1	PE	Bio-Rad	MCA5954PE
CD14	TÜK4	Human	Mouse	IgG2a	APC-Vio770	Miltenyi Biotec	130-113-144
CD14	MIL2	Pig	Mouse	IgG2b	PE	Bio-Rad	MCA1218F
CD16	G7	Pig	Mouse	IgG1	FITC	ThermoFisher	MA1-80267
CD163	2A10/11	Pig	Mouse	IgG1	-	ThermoFisher	MA516476
CD172a/SWC3/ Monocyte/ Granulocyte	74-22-15A	Pig	Mouse	IgG2b	PE	BD Biosciences	561499
CD172a/SWC3/ Monocyte/ Granulocyte	74-22-15	Pig	Mouse	IgG1	Biotin	Southern Biotech	SBA-4525-08
CD203a/SWC9	PM18-7	Pig	Mouse	IgG1	-	Bio-Rad	MCA1973GA
CD206	19.2	Human	Mouse	IgG1	APC	eBioscience	17-2069-42
FoxP3	FJK-16S	Human	Rat	IgG2a	PerCP-Cy5.5	eBioscience	45-5773-82
GATA-3	TWAJ	Human	Rat	IgG2B	PE	eBioscience	12-9966-42
IFNg	P2G10	Pig	Mouse	IgG1	Alexa 647	BD Biosciences	561480
IgG1	RMG1-1	Mouse	Rat	IgG	Alexa 700	BioLegend	406632
IgG1	RMG1-1	Mouse	Rat	IgG	APC-Cy7	BioLegend	406620
IgG1	RMG1-1	Mouse	Rat	IgG	PE-Cy7	BioLegend	406613
IgG2a	R19-15	Mouse	Rat	IgG1	Brilliant Violet 605	BD Biosciences	564024
IgG2a	RMG2a-62	Mouse	Rat	IgG	PerCP-Cy5.5	BioLegend	407112
IgG2b	RMG2b-1	Mouse	Rat	IgG1, κ	FITC	BioLegend	406706
IgG2b	Polyclonal	Mouse	Goat	-	APC-Cy7	Southern Biotech	SBA-1090-19
IL-4	MP4-25D2	Human	Rat	IgG1	PE-Cy7	BioLegend	500824
IL-17a	eBio64DEC17	Human	Mouse	IgG1	FITC	eBioscience	11-7179-82
SLAII-DR	2E9/13	Pig	Mouse	IgG2b	-	ThermoFisher	MA5-28503
SLAII-DR	2E9/13	Pig	Mouse	IgG2b	FITC	Bio-Rad	MCA2314F
Streptavidin	-	-	-	-	PerCP-Cy5.5	BioLegend	405214

Streptavidin	-	-	-	-	Alexa 700	ThermoFisher	S21383
T-bet	4B10	Human	Mouse	IgG1	Brilliant Violet 605	BioLegend	644817
TCR1 δ	PGBL22A	Pig	Mouse	IgG1	-	Kingfisher	WS0621S-100
TNFa	MAb11	Human	Mouse	IgG1	APC-Cy7	BioLegend	502944

Table S2. Primers used in this study

Gene	Primer	Sequence (5'-3')
IL8	Forward	TTCGATGCCAGTGCATAAATA
	Reverse	CTGTACAACCTTCTGCACCCA
CCL2	Forward	ACCAGCAGCAAGTGTCCTAAAG
	Reverse	GTCAGGCTTCAAGGCTTCGG

Table S3. Total Cell Counts from Organ Samples Used for Assessing Frequencies of Macrophage and Monocyte-Macrophage Populations in Figures 4 and 6, respectively.

	Total Cell Count (x10^8)			
**Pig**	**Jejunum LP**	**Ileum LP**	**Liver**	**Lung**
ST 1	4.20	1.98	1.20	1.68
ST 2	1.08	0.56	1.24	0.90
ST 3	0.96	0.48	1.80	1.56
ST 4	1.86	0.45	6.00	3.90
ST 5	1.08	0.52	7.60	6.60
ST 6	1.11	1.68	12.0	1.20
As+ST 1	3.81	0.59	2.00	2.28
As+ST 2	3.56	0.49	3.60	2.22
As+ST 3	0.51	0.15	2.00	2.41
As+ST 4	0.49	0.70	9.00	2.92
As+ST 5	0.17	0.69	3.80	1.29
As+ST 6	2.76	0.60	4.80	2.33
As 1	0.64	0.46	6.5	1.00
As 2	0.71	0.40	9.9	3.20
As 3	1.84	1.16	10.0	2.00
As 4	1.80	1.10	9.6	4.60
As 5	0.57	0.18	1.9	1.20
As 6	0.32	0.64	9.1	1.06
Ctrl 1	1.74	0.72	8.00	2.05
Ctrl 2	0.72	0.24	6.80	2.01
Ctrl 3	0.92	0.31	5.3	1.46
Ctrl 4	1.62	0.35	5.4	2.00
Ctrl 5	0.61	0.54	2.00	1.29
Ctrl 6	0.96	1.50	1.40	1.66
***P*-value**	0.4493	0.8431	0.2238	0.5470

Differences in total cell counts assessed by Kruskal-Wallis test.

References Cited in Supplemental Material

1. Midha A, Jarquín-Díaz VH, Ebner F, Löber U, Hayani R, Kundik A, Cardilli A, Heitlinger E, Forslund SK, Hartmann S. 2022. Guts within guts: the microbiome of the intestinal helminth parasite *Ascaris suum* is derived but distinct from its host. Microbiome 10:229. https://doi.org/10.1186/s40168-022-01399-5

2. Scherer K, Szabó I, Rösler U, Appel B, Hensel A, Nöckler K. 2008. Time course of infection with *Salmonella* typhimurium and its influence on fecal shedding, distribution in inner organs, and antibody response in fattening pigs. J Food Prot 71:699–705. https://doi.org/10.4315/0362-028X-71.4.699

3. Cossarizza A, Chang H-D, Radbruch A, Acs A, Adam D, Adam-Klages S, Agace WW, Aghaeepour N, Akdis M, Allez M, et al. 2019. Guidelines for the use of flow cytometry and cell sorting in immunological studies (second edition). European Journal of Immunology 49:1457–1973. https://doi.org/10.1002/eji.201970107

4. Siegmund B, Lear-Kaul KC, Faggioni R, Fantuzzi G. 2002. Leptin deficiency, not obesity, protects mice from Con A-induced hepatitis. European Journal of Immunology 32:552–560. https://doi.org/10.1002/1521-4141(200202)32:2<552::AID-IMMU552>3.0.CO;2-H

5. Perdomo C, Zedler U, Kühl AA, Lozza L, Saikali P, Sander LE, Vogelzang A, Kaufmann SHE, Kupz A. 2016. Mucosal BCG Vaccination Induces Protective Lung-Resident Memory T Cell Populations against Tuberculosis. mBio 7. https://doi.org/10.1128/mbio.01686-16

6. Erben U, Loddenkemper C, Doerfel K, Spieckermann S, Haller D, Heimesaat MM, Zeitz M, Siegmund B, Kühl AA. 2014. A guide to histomorphological evaluation of intestinal inflammation in mouse models. Int J Clin Exp Pathol 7:4557–4576.

7. Livak KJ, Schmittgen TD. 2001. Analysis of relative gene expression data using real-time quantitative PCR and the 2(-Delta Delta C(T)) Method. Methods 25:402–408. https://doi.org/10.1006/meth.2001.1262
